# Redundancy of primary RNA-binding functions of the bacterial transcription terminator Rho

**DOI:** 10.1093/nar/gku690

**Published:** 2014-07-31

**Authors:** Rajesh Shashni, M. Zuhaib Qayyum, V. Vishalini, Debashish Dey, Ranjan Sen

**Affiliations:** Laboratory of Transcription, Center for DNA Fingerprinting and Diagnostics, Tuljaguda Complex, 4-1-714 Mozamjahi Road, Nampally, Hyderabad 500001, India

## Abstract

The bacterial transcription terminator, Rho, terminates transcription at half of the operons. According to the classical model derived from *in vitro* assays on a few terminators, Rho is recruited to the transcription elongation complex (EC) by recognizing specific sites (*rut*) on the nascent RNA. Here, we explored the mode of *in vivo* recruitment process of Rho. We show that sequence specific recognition of the *rut* site, in majority of the Rho-dependent terminators, can be compromised to a great extent without seriously affecting the genome-wide termination function as well as the viability of *Escherichia coli*. These terminators function optimally only through a NusG-assisted recruitment and activation of Rho. Our data also indicate that at these terminators, Rho-EC-bound NusG interaction facilitates the isomerization of Rho into a translocase-competent form by stabilizing the interactions of mRNA with the secondary RNA binding site, thereby overcoming the defects of the primary RNA binding functions.

## INTRODUCTION

The well-conserved bacterial transcription terminator, Rho, is a hexameric RNA helicase that terminates transcription at about half of the operons (1,2). Rho-dependent termination process is essential to stop unwanted transcription in the events of uncoupling of transcription and translation (1) in suppressing anti-sense or erroneous transcription (3) and in modulating riboswitch formation (4). This process is involved in gene regulation by small RNAs (5), in silencing horizontally transferred genes (6), required for prophage maintenance (7) and, finally, is also speculated to prevent R-loop formation (8).

NusG, a transcription elongation factor, interacts with Rho through its C-terminal domain (CTD), and facilitates the transcription termination process (9). Exact mechanism of this facilitation process is not known. *In vitro*, NusG induces early termination and increases the speed of RNA release, and thereby is thought to function in the RNA release step of Rho-dependent termination (10–12).

According to the classical model, Rho at first recognizes specific sites on the mRNA, called *rut* sites (Rho utilization), using its primary RNA binding sites (PBSs). Following that, it isomerizes into a translocase-competent form by threading the mRNA through its secondary RNA binding sites (SBSs), undergoes ATP-hydrolysis-dependent translocation along the mRNA and, finally, dislodges the elongation complex (EC; Supplementary Figure S1, the RNA-dependent pathway; 1,2). Recently, in an alternative model, Rho has been proposed to be recruited on the surface of the RNAP prior to the interaction with the mRNA (RNAP-dependent pathway; Supplementary Figure S1; 13,14). Genome-wide ChIP analyses have indicated that Rho remained associated with the RNAP throughout the transcription cycle (13), and a set of *in vitro* experiments also described the association of Rho with the RNAP, following which it gets transferred to the mRNA (14).

In our previous work, using direct *in vitro* nascent RNA-binding assays, we have shown that on a strong terminator, *trpt′*, Rho can only be associated with the ECs having longer nascent (>100 nt) RNAs comprising of *rut* sites (15). Here, we explore the *in vivo* regulations involved in the recruitment of Rho to the EC by comparing the behaviors of Rho mutants defective for the PBS (PBS mutants) and the SBS functions (SBS mutants). The effect of these mutants on the genome-wide transcription pattern revealed that primary RNA binding function of Rho, unlike ATPase and translocase activities, can be compromised in majority of the terminators including those in the rac (*t*_rac__;_ Supplementary Figure S3) and other prophages, whose suppression by Rho is essential for the cell viability (6). However, in the purified system, the PBS mutant was significantly defective at *t*_rac_, which led us to hypothesize an assisted recruitment model of Rho to the EC at these terminators *in vivo*. Unusually high NusG dependence of termination at *t*_rac_, specific synthetic lethality of PBS mutants in the presence of NusG CTD mutants (defective for Rho-binding) and slow RNA-induced isomerization of Rho at the *rut* sites of *t*_rac_ strongly indicated that Rho-EC bound NusG interactions act during the recruitment of Rho to the EC and provide additional stability to the Rho–mRNA complex at the weak *rut* sites of many terminators.

## MATERIALS AND METHODS

### Materials

Nucleoside triphosphates (NTPs) for *in vitro* transcription reactions were purchased from GE healthcare. [γ-^32^P]ATP (3000 Ci/mmol) and [α-^32^P]CTP (3000 Ci/mmol) were from Jonaki, BRIT, India. Antibiotics, isopropyl β-D-1-thiogalactopyranoside (IPTG), lysozyme, dithiothreitol (DTT) and bovine serum albumin (BSA) were from USB. Primers for polymerase chain reaction (PCR) were obtained either from Sigma or MWG. Restriction endonucleases, polynucleotide kinase and T4 DNA ligase were from New England Biolabs. WT *Escherichia coli* RNAP holoenzyme was purchased from Epicentre Biotechnologies. Taq DNA polymerase was from Roche Applied Science. Ni-NTA agarose beads were from Qiagen. Streptavidin-coated magnetic beads were from Promega. Bacterial RNA purification kit was from NEB. RNAlater^TM^ used for storing RNA samples used in microarray experiments were from Ambion. All the bacterial growth media were from Difco.

### Strains, plasmids, phages, etc.

Scar-less deletions (removal of *kan^R^* cassette from *rho*; *rho::FRT*) of chromosomal *rho* (strains RS1458 and RS1490) have been made by expressing flip recombinase from a temperature-sensitive (‘ts’) plasmid, pRS766. Subsequently, the plasmid was removed by growing the strains at 42°C. *nusG* mutations, G146D and L158Q, were inserted into the chromosome by linear DNA transformation. In brief, the strain RS1428 was transformed with PCR-amplified DNA fragments containing the mutations. RS1428 expresses *λ* red recombinase from a ‘ts’ plasmid and has a chromosomal insertion of a *t*_rac_*-LacZYA* reporter cassette (as a λRS45 lysogen). The recombinants were screened on minimal media containing lactose. Colonies with *nusG* mutants was able to read through the *t*_rac_ terminator and thereby ensured the cells to utilize lactose. Presence of *nusG* mutants was further confirmed by suppression of the phenotype by expressing WT *nusG* (pRS695) and sequencing.

All the bacterial strains and plasmids are listed in Table 1. List of different oligos used in this study is given in Supplementary Table S1.

**Table 1.** List of strains and plasmids used

Strains	Description	Reference
RS1263	MG1655 WT strain	
RS862	MG1655 WT Δ*rac* strain	This study
RS257	MC4100 *galEP3* Δ*rac*(GJ3161)	J. Gowrishankar (8)
RS330	RS257 made *Δrho::* Kan^R^ with pHYD1201, Amp^R^	J. Gowrishankar (8)
RS709	RS257 with λRS45 lysogen carrying P*_lac_*-H19B *tR1 -lacZYA*, *Δrho::* Kan^R^ with pHYD1201, Amp^R^	Chalissery *et al.* (11)
RS734	RS257 with λRS45 lysogen carrying P*_lac_*-H19B *tR1* -*lacZYA* (GJ5147)	J. Gowrishankar (16)
RS1428	RS257 with λRS45 lysogen carrying P*_RM_*-*racR*-*t_rac_*-*lacZYA*	This study
RS1429	RS1428 made *Δrho::* Kan^R^ with pHYD1201, Amp^R^	This study
RS1430	RS 1429 Δ*rho*::Kan^r^ made marker less *Δrho::*FRT with pHYD1201, Amp^R^	This study
RS1458	RS257 with λRS45 lysogen carrying P*_lac_*-H19B *tR1*-*trpt’*-*lacZYA*,Δ*rho*(marker less, *Δrho::* FRT) with pHYD1201, Amp^R^	This study
RS1490	MC4100 *galEp3* with λRS45 lysogen carrying P*_lac_*-H19B *tR1-lacZYA*, Δ*rho*(marker less, *Δrho::* FRT) with pHYD1201, Amp^R^	This study
RS1514	RS1428 with G146D *nusG* in chromosome	This study
RS1523	RS1428 with L158Q *nusG* in chromosome	This study
Plasmids		
pHYD1201	3.3 kb *Hin*dIII-*Sal*I fragment carrying *rho*^+^subcloned from pHYD567 into *Hin*dIII/*Sal*I sites of pAM34 (pMB1; IPTG-dependent replicon, Amp^R^)	Harinarayanan and Gowrishankar, 2003 (8)
pRS22	pTL61 T with pT7A1-*H-19B nutR-T_R′_T_1_T_2_-lacZYA*, Amp^R^	Cheeran *et al.* (17)
pRS106	pT7A1*-trpt′-lacZ*,Amp^R^	Pani *et al.* (18)
pRS346	pHYD567-*rho* N340S, Sp^R^, Sm^R^	Chalissery *et al.* (11)
pRS350	pHYD567-*rho* Y80C, Sp^R^, Sm^R^	Chalissery *et al.* (11)
pRS567	λ red recombinase under pBAD promoter subcloned from pKD46 in pCL1920 (pSC101), Sp^R^, Sm^R^	Shashni *et al.* (19)
pRS604	T7A1-λ*T_R_*_1_ fragment cloned at HindIII site of pRS22.	Dutta *et al.* (20)
pRS649	pHYD567-*rho* WT, Sp^R^, Sm^R^	Chalissery *et al.* (11)
pRS695	pHYD3011 WT S60A *nusG*, Amp^R^	Chalissery *et al.* (12)
pRS700	pHYD3011 S60A G146D *nusG*, Amp^R^	Chalissery *et al.* (12)
pRS728	pHYD3011 S60A L158Q *nusG*, Amp^R^	Chalissery *et al.* (12)
pRS729	pHYD3011 S60A V160N *nusG*, Amp^R^	Chalissery *et al.* (12)
pRS766	pcp20 (ts), *Flp recombinase, Amp*^R^, *Cam*^R^	Cherepanov *et al.* (26)
RS1246	pHYD567-*rho* F62S, Sp^R^, Sm^R^	This study
pRS1352	(P*_RM_*-*racR*-*t_imm_*-*lacZYA*) cloned at EcoRI-SmaI of pRS19 (pK8628), Amp^R^	This study

### Cell growth assays

**Figure 1.**
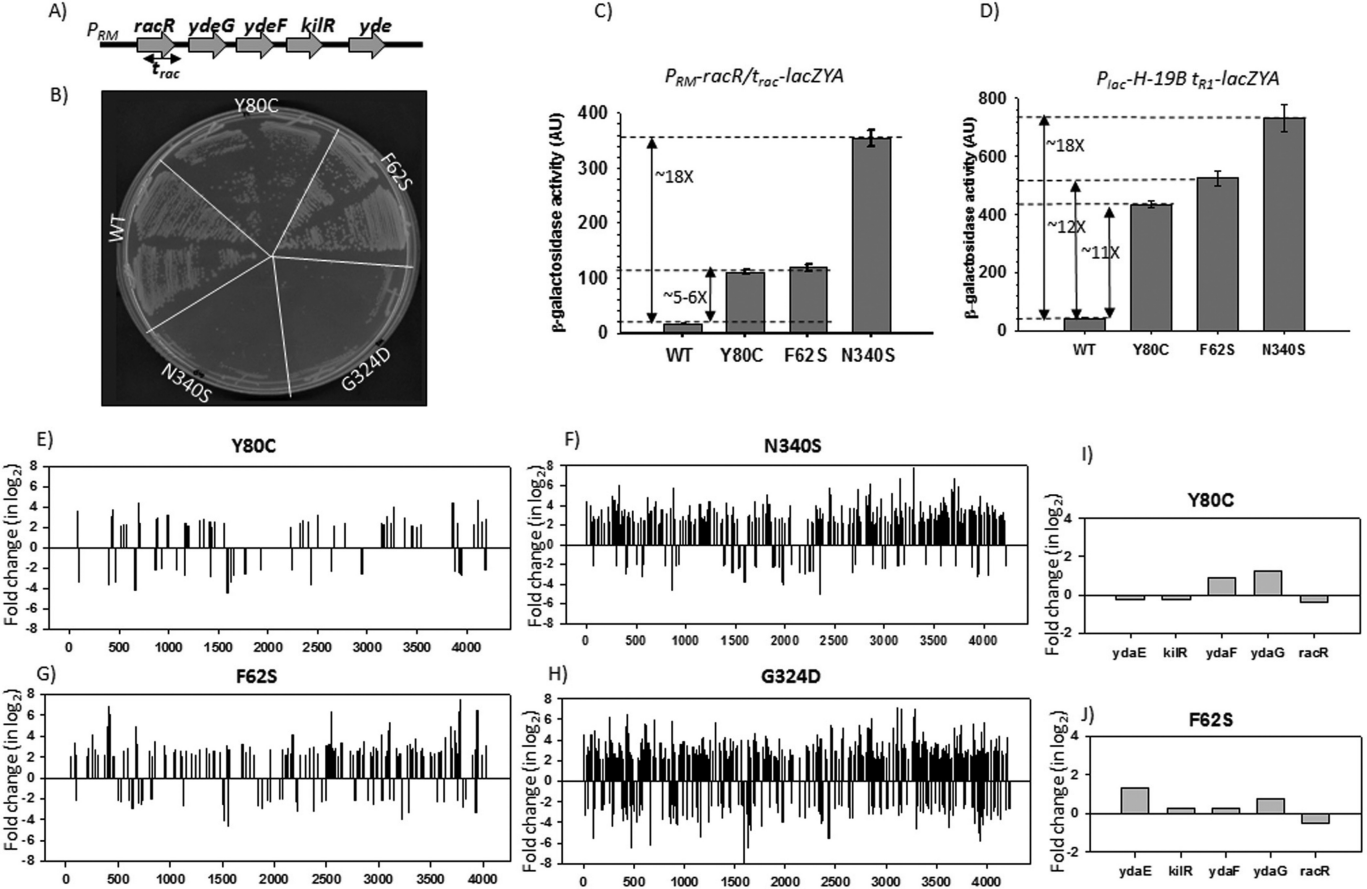
(**A**) Gene organization in the rac prophage region. Location of the *t*_rac_ terminator is indicated. Expression of *kilR*, an inhibitor of Ftz, is lethal to the cells. In (**B**), growth of *MG1655rac^+^Δrho::kan^R^* strains in the presence of different *rho* mutants expressed from a low copy plasmid pCl1920 is shown. Two transductants of each *rho* mutant from the P1 transduction plates, as shown in Supplementary Figure S4A, were re-streaked on LB plates. (**C**) and (**D**) β-galactosidase activities of MC4100 *rac^−^Δrho::kan^R^* strains harboring pCl1920 plasmids expressing different PBS and SBS mutants. In (**C**), the *lacZ* reporter cassette was fused downstream of *t_rac_* terminator, and in (**D**), it was fused to *t*_R1_ terminator. Both the reporters were present as λRS45 lysogens. Error bars were calculated from the values obtained from six independent colonies. Fold changes in the activity with respect to the WT are indicated. (**E**)–(**H**) Plots of microarray profiles obtained from MG1655*rac^+^* (for PBS) and MG1655*rac^−^* (for SBS) strains expressing different *rho* mutants as indicated. The ratio of the hybridization intensities obtained from WT and different mutants gave the measure of fold change that is expressed in log_2_ scale as per convention. ‘+’ fold changes denote up-regulation, whereas ‘–’ fold changes denote down-regulation of the genes. In (**I**) and (**J**), expressions of the genes in the rac prophage region were highlighted for the two PBS Rho mutants. Data were taken from (E) and (F).

To study the differential growth behavior between the WT and different *rho* mutants (Figure 1), MG1655*rac^+^*strains (having the rac prophage) were at first transformed with WT and different Rho mutants expressed from a low copy number plasmid, pCL1920 (pHYD567), and, subsequently, the chromosomal *rho* was removed by inserting a *kan^R^* cassette (*rho::kan^R^*) via P1 transduction (Supplementary Figure S4A). The transductants were re-streaked on the LB plates supplemented with appropriate antibiotics (Figure 1B). In this *rho::kan^R^* cassette, first 360 bases of the *rho* sequence are replaced with the kanamycin resistance gene, and hence *rho* is linked very tightly with this marker (P1 was made on the strain RS330; see Table 1). We have also confirmed the absence of duplication of *rho* in all the transductants by PCR using *rho* specific primers (Supplementary Figure S4B).

### Synthetic lethality assays

For these assays, we have used MC4100 strains that have lost rac prophage (*rac^−^*) during the propagation, and deletion of this prophage allowed the strain to survive in the presence of all the *rho and nusG* mutants. This MC4100 also has the *t*_rac_ terminator cassette inserted into the genome as a λRS45 lysogen (RS1428). *nusG* mutations were then inserted into the genome of RS1428 by linear DNA transformations to produce RS1514 (G146D) and RS1523 (L158Q). These two resultant strains were transformed with WT and different Rho mutants (Y80C and N340S) cloned in a low copy number plasmid, pCL1920 (pHYD567), and subsequently the chromosomal *rho* was removed by transducing a *rho::kan^R^* cassette via P1 transduction. Two transductants of each mutants were re-streaked to measure the synthetic lethality (Figure 3F).

**Figure 2.**
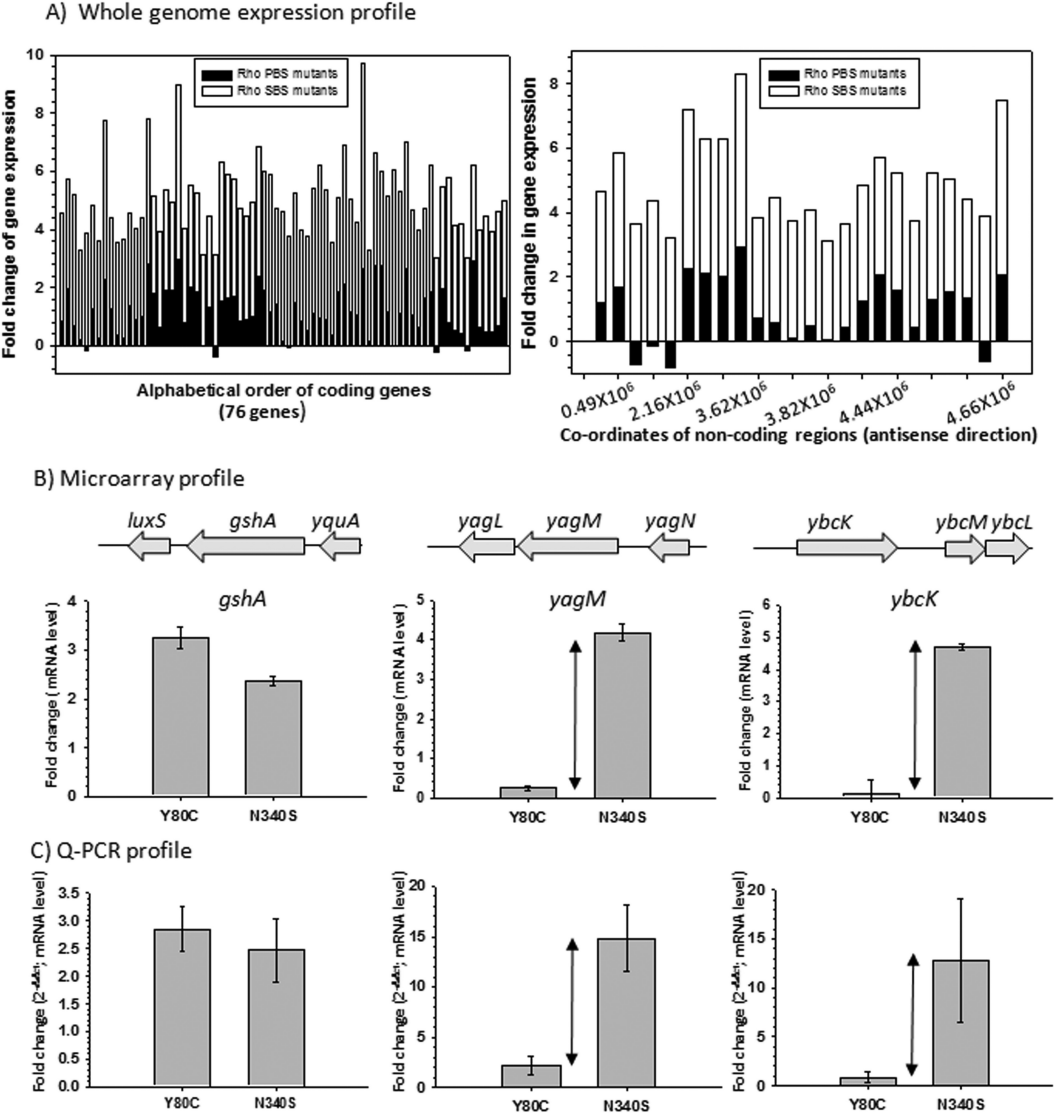
(**A**) Fold changes in gene expression for the genes that are less affected in PBS mutants were overlaid onto the fold changes in gene expression of the same genes obtained in the presence of SBS mutants. In these plots, mean values of fold changes of two different PBS mutants (Y80C and F62S) were overlaid onto the mean values obtained from N340S and G324D SBS mutants. Left panel depicts data from coding genes, whereas the right panel is for non-coding regions along the antisense direction. (**B**) Validation of the microarray data of the selective genes by qRT-PCR. Upper panel shows the microarray profiles of the indicated genes in the presence of Y80C and N340S Rho mutants. Bottom panel validates the expression profiles of the same genes using qRT-PCR. Fold changes in qRT-PCR were expressed in terms of C_T_ values (threshold cycle) as per the convention, details of which are given in the Materials and Methods section. Error bars were calculated from the data obtained from RNA preparations of two independent colonies. Double-sided arrows indicate the differences in gene expression levels in the presence of PBS and SBS mutants.

**Figure 3.**
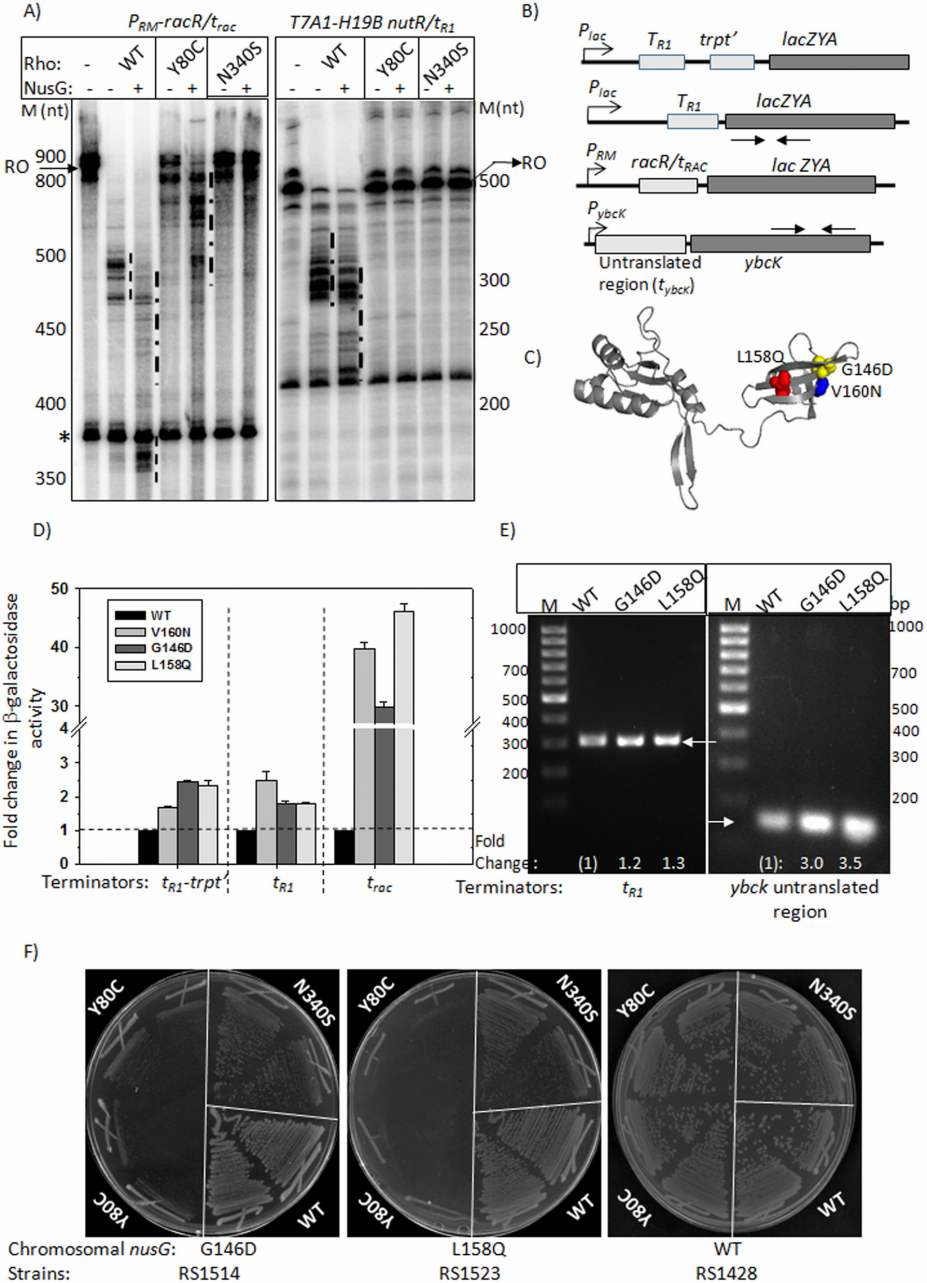
(**A**) Autoradiograms showing the *in vitro* transcription termination assays on two types of linear DNA templates having *t*_rac_ and *t*_R1_ terminators under different conditions as indicated. Run-off (RO) products and terminated products (dashed lines) are indicated. ‘*’ is an arrested product characteristic of this template. Molecular weight markers (M) are indicated adjacent to the autoradiograms. (**B**) Schematic showing constructs where different terminators, *t*_R1_*, trpt′* and *t*_rac_, were fused upstream of the *lacZ* reporter. An untranslated region, having putative Rho-loading site(s) (*t*_ybck__;_ see Supplementary Figure S8A), is located upstream of *ybcK*. *LacZ* reporter cassettes were inserted in the chromosome of MC4100 strain by λRS45 mediated transduction. Arrows indicate primer pairs used in RT-PCR reactions. (**C**) NusG-CTD point mutants defective for Rho-binding are shown on the structure (12). (**D**) Bar diagrams showing the β-galactosidase activities obtained from the indicated terminator*-lacZ* fusions in the presence of WT and different NusG mutants expressed from pHyd311 plasmid. Y-axis has been shown as a broken scale to accommodate the full range of values. Errors were obtained by measuring the activities of six independent colonies. The activities in the presence of WT NusG were set at 1, and the fold change values in the presence of NusG mutants were expressed with respect to the WT value. The raw data of β-galactosidase activities are described in Supplementary Table S2. (**E**) EtBr stained 1.5% agarose gel showing the RT-PCR products obtained from the *lacZ* gene fused to *t*_R1_ terminator (left panel) and from the *ybcK* (right panel) using appropriate primer pairs indicated in (B). RNA was isolated from the strains expressing indicated WT or mutant NusG supplied from pHYD311. In a similar way as above, the amount of RT-PCR products (intensity of the bands) was also expressed as fold change with respect to WT NusG. Intensities of the bands were measured using Image J, a version of NIH Image for Personal Computers (PCs). (**F**) Growth characteristic of MC4100*rac^−^Δrho nusGG146D* or *nusGL158Q* or *WT nusG* upon transformations with pCL1920 plasmids expressing either WT or N340S or Y80C Rho mutants. Two transductants of each strain were re-streaked on LB plates.

### *In vivo* termination assays

All the *in vivo* termination assays were performed in different derivatives of *E. coli* MC4100*Δrho rac*^−^ strains, where Rho is supplied from a shelter plasmid, pHYD1201. We used strains devoid of rac prophage (rac^−^) so that they are less dependent on Rho-dependent termination for their survival (6) and, therefore, are viable in the presence of all the *rho* mutants that we have used. These strains used for β-galactosidase assays contain single-copy, *P*_lac_*-H-19B t*_R1_*-trpt′-lacZYA* (RS1458), *P*_lac_*-H-19B t*_R1_*-lacZYA* (RS1490) and *P*_RM_*-racR/t*_rac_*-lacZYA* (RS1430) reporter cassettes as λRS45 lysogens. In all these reporters, expression of lacZ occurs only in the absence of Rho-dependent termination, and hence was used for measuring the termination defects (Figures 1C and D and 3D). These strains were transformed with WT or different mutant derivatives of Rho expressed from pCL1920, and this process replaced the shelter plasmid expressing the *rho*.

To study the effects of *nusG* mutants (Figure 3D), the strains RS1490, RS1458 and RS1430 were transformed with the pHYD3011 plasmids expressing mutant and WT NusG. In these strains, WT Rho was expressed from a low copy pCL1920 plasmid. The β-galactosidase activities were measured using a SpectraMax Plus microtiter plate reader (11).

*In vivo* transcription of the chromosomal *ybcK* gene in the presence of different *nusG* mutants was measured by real-time PCR (RT-PCR). Total RNA was isolated from MC4100 (RS734) strain transformed with pHYD3011 plasmid expressing either WT or the *nusG* mutants, L158Q and G146D. RT-PCR reactions were performed on two genes; *lacZ*, fused downstream of *t*_R1_ terminator, and *ybcK*, which follows an untranslated region likely to have Rho-binding site(s) (Supplementary Figures S8A and S10A). The primer pairs RS401/402 and RS848/849 (see Supplementary Table S1) were used to amplify the cDNA products made from the RNA transcripts of *lacZ* and *ybcK* genes, respectively. PCR products were visualized in a 1.5% agarose gel stained with EtBr (Figure 3E).

To measure the *in vivo* termination zone inside the *racR* (Supplementary Figure S12), RS1428 was transformed with either WT or N340S Rho mutants cloned in pCL1920 plasmid and the genomic copy of *rho* was deleted by P1 (*Δrho:kan^R^*) transduction. In all the RT-PCR assays, total RNA was isolated from these strains using RNeasy RNA isolation kit (Qiagen). cDNA was synthesized from 2 μg of isolated RNA using SuperScript III First Strand Synthesis System (Invitrogen Inc.) as per the manufacturer's protocol. cDNA was used as a template in RT-PCR with different oligos designed inside the gene of interest (Supplementary Table S1 and Supplementary Figures S8 and S12). To identify the *in vivo* Rho-dependent termination events in the *t*_rac_ terminator region, we compared the amounts of RT-PCR products obtained from the strains expressing WT and N340S Rho mutant. Higher amount of PCR products in N340S lanes (Supplementary Figure S12) is due to impaired termination events.

### Microarray analyses

The *MG1655* strain was at first transformed with pCL1920 plasmids containing WT and different Rho mutants, and the chromosomal *rho* was deleted (*rho::kan^R^*) by P1 transduction. For the PBS mutants, *MG1655rac^+^*, and for the SBS mutants, *MG1655rac^−^* strains were used. Deletion of rac prophage (*rac*^−^) allows the strains to grow in the presence of SBS mutants. Overnight cultures of these strains were sub-cultured in 10 ml LB with appropriate antibiotics, and were allowed to grow until OD_600_ ∼ 0.3–0.4. Culture was spun down and the cell pellet was re-suspended in 1 ml of RNAlater ^TM^.

RNA isolation and the microarray experiments were performed by Genotypic Technology, Bangalore, India. In brief, RNA was extracted from the cell pellets suspended in RNAlater solution using Qiagen's RNeasy kit. Agilent *E. coli* K12 microarray array slides in a 8 × 15k format were used. Agilent's two-color microarray-based gene expression analysis uses cyanine 3- and cyanine 5-labeled targets to measure gene expression in experimental and control samples. The total number of coding regions available on the chip was 4294. The average number of probes per coding region sequence was 3, and the total number of probes designed was 10 828. The total number of probes used for non-coding region was 4380. Two independent biological replicates for each strain were taken for the analyses. Fold change in gene expression for each strain was calculated with respect to WT. We have considered the genes whose expressions were changed 5-fold (log 2) or more with respect to the WT.

### qRT-PCR analyses

The total RNA used in the quantitative RT-PCR (qRT-PCR) experiments was prepared in the same way as for microarray experiments. The amount of cDNA produced during the PCR cycles was monitored using SYBR green dye in an Applied Biosystem 7500 PCR system. C_t_, the number of threshold cycle (the midpoint of the sigmoidal curve obtained by plotting the fluorescence intensity against the number of PCR cycles), was used to quantitate the amount of cDNA. We have used the following mathematical equations to express the amount of the qRT-PCR products. 2^−ΔΔCt^ method was used to calculate the fold change (2^−ΔΔCt^) in the mRNA level for the mutants with respect to the WT, where C_t_ = number of threshold cycle; ΔCt = Ct of target gene – Ct of internal control; ΔΔCt = ΔCt of the mutant – ΔCt of the WT. The level of *rpoC* mRNA was independent of the Rho mutants and, therefore, it was used as an internal control. Primer pair for each gene was chosen based on the following criteria: (i) comparable T_M_ between the primers, (ii) located at the central part of the gene and (iii) the length of the cDNA product is ∼200–300 nt.

### Templates for *in vitro* transcriptions

Linear DNA templates for *in vitro* transcription assays were made by PCR amplification from the plasmids, pRS22 (T7A1-H-19B *nutR/t*_R1;_ oligo pairs RS83/RS404 and RS83/RK23B), pRS106 (pT7A1-*trpt′*; oligo pair RS83/RSRK-1), pRS604 (pT7A1-*λt*_R1_-T_1_T_2_; oligo pair RS83/RS147) and pRS1352 (P_RM_-*racR/t*_rac_; oligo pairs RS83/RSRK-1, RS83/RS845, RS83/RS956 and RS83/RS955). When required, a lac operator sequence was inserted either after *t*_R1_ or *t*_rac_ terminators using a downstream primer having the operator sequence. In pRS385, the lac operator sequence is cloned after the H-19B *t*_R1_ terminator (15). In order to immobilize the DNA templates to the streptavidin-coated magnetic beads, a biotin group at the 5′-end of the templates was incorporated by using the biotinylated primer RS83. In aforementioned templates, transcription was initiated from T7A1 or a P_RM_ promoter.

To construct the linear DNA template for *in vitro* transcription of the *yagNM* operon, PCR amplification was performed using the primer RS1083/RS1084 pairs on the genomic DNA prepared from the MG1655 strain. In this template, transcription is directed from one of the promoters indicated in Supplementary Figure S8B.

All the plasmids and oligos used for *in vitro* transcription assays are listed in Supplementary Table S1.

### *In vitro* transcription assays

For the transcription using strong T7A1 promoter, we followed the regime of single-round transcription. However, due to the poor yield from P_RM_ promoter and the natural promoters (P_yag_) of *yagNM* operon, multiple-rounds transcription was employed by omitting rifampicin from the reaction mixture (Figure 3A and Supplementary Figure S11). Reactions were carried out in transcription buffer (20 mM Tris-Cl, pH 8.0, 10 mM Mg-Cl, 50 mM K-Cl, 1 mM DTT and 100 μg/ml BSA) at 37°C. For single-round transcription, the reactions were initiated with 175 μM ApU, 5 μM GTP, 5 μM ATP, 2.5 μM CTP and [α-^32^P]CTP (3000Ci/mmol; BRIT, Jonaki, Hyderabad) to make a 23-mer EC (EC_23_). The EC_23_ was then chased with 250 μM each of ATP, GTP, CTP and UTP. For multiple-round transcription, the RNAP–promoter complex was chased with 250 μM each of ATP, GTP and UTP. Concentration of CTP was 50 μM and the RNA was labeled with [α-^32^P]CTP (3000Ci/mmol). The reactions were stopped by extraction with phenol followed by ethanol precipitation. Samples were loaded onto a 6% sequencing gel and analyzed using FLA 9000 phosphorimager (Fuji).

For measuring the RNA release kinetics from the stalled EC, transcription reactions were performed in presence of 100 nM lac repressor so that the EC gets road-blocked at the *lacO* site present downstream of the terminator region. The DNA templates were immobilized on streptavidin-coated magnetic beads (Promega) through streptavidin–biotin interaction. The transcription reactions were continued for 3 min to form the stalled EC, and the excess NTPs were removed by thoroughly washing the beads, following which 50 nM Rho plus 1 mM ATP was added. Ten microliters of samples were removed at different time points, and separated into supernatant (S) and pellet (P) on magnetic stands. Half of the supernatant (S) was directly mixed with equal volume of formaldehyde loading buffer. Rest of the sample (S+P) was phenol extracted and mixed with dye. Fraction of released RNA [(2S)/(S) + (S+P)] was plotted against time and the curves were fitted to exponential rise equations either of the form *y* = *a*(1−exp^−*bx*^) or *y* = *y*_0_ +*a*(1−exp^−*bx*)^, where ‘*b*’ denotes the rate, ‘*a*’ is amplitude of the RNA release process and ‘*y*_0_’ is minimum value of *y* at 0 time point. RNA release kinetics on *t*_rac_ terminator had a distinct lag period and was fitted to a sigmoidal equation of the form: *y* = *y*_0_ + (*a*)/[1 + (*x*/*x*_0_)^*b*^], where ‘*x*_0_’ is the inflection point of the curve and ‘*b*’ is the slope factor.

### ATPase assays

We used the nascent RNA attached to the stalled ECs to activate the ATPase function of Rho (22). The stalled ECs were formed in a similar way as in the RNA release kinetics of Rho but without using streptavidin magnetic beads. DNA templates were prepared by PCR amplification from the plasmid pRS22 using RS83/RS404 oligo pair and from the plasmid pRS1352 using RS83/RS955 oligo pair to make nascent RNA containing a *t*_R1_ or a *t*_rac_ sequence, respectively. To get adequate amount of RNA for the ATPase assays, the concentrations of DNA template and RNAP were increased to 4-fold compared to that used in the *in vitro* transcription assays. On these templates, road-blocked (RB) complexes were formed in a similar way as in RNA release kinetics assays. Due to the weaker transcription initiation efficiency of P_RM_ promoter fused to the *t*_rac_, compared to that of the T7A1 promoter fused to *t*_R1_, two reactions using the former fusion were pooled together and concentrated through Amicon-100 columns to get the desired volume. Rho + 1 mM ATP duped with [γ-^32^P]-ATP (3000Ci/mmole) was added to the RBs, and the time course of inorganic phosphate (Pi) release (ATP hydrolysis) was monitored on polyethyleneimine (PEI) TLC sheets (Merck) with 0.75 M KH_2_PO_4_ (pH 3.5) as the mobile phase. In all the ATPase assays, the composition of the reaction mixtures was 25 mM Tris-HCl (pH 8.0), 50 mM KCl and 5 mM MgCl_2_. The reactions at indicated time points were stopped with 1.5 M formic acid. TLC sheets were analyzed by Fuji FLA-9000. The amounts of [γ-32P] ATP and the released Pi were calculated from the intensities of the spots of these products using the Image-Quant software.

## RESULTS

### Rationale

The Rho protein has two RNA binding sites, PBS and SBS, with distinct functions (1). To monitor the involvement of these two sites in the Rho-recruitment process *in vivo*, we have chosen specific Rho mutants having changes in these two sites (Supplementary Figure S2A). PBS mutants are defective for *rut* site recognition (Supplementary Figure S2B), while SBS mutants can interact with RNA at the PBSs but have impaired RNA-dependent ATPase activity and, consequently, are defective in translocation along the mRNA (Supplementary Figure S2C; 11,23). We have chosen two PBS mutants, Y80C and F62S, both of which are extremely defective in binding to RNA (Supplementary Figure S2B; 11,23). We compared these mutants with the SBS mutants, N340S and G324D. These mutants do not have significant defect in RNA recognition, but are defective for ATPase as well as translocase activities (11). In order to decipher the necessity of the *rut* site recognition *in vivo*, we measured the termination behaviors of these mutants on specific terminators and also monitored their effects on the genome-wide transcription termination profiles.

### Rho-dependent termination in the rac prophage genes depends on the nature of the Rho mutants

One of the major functions of Rho-dependent termination shown was to suppress the transcription from foreign genes, like those in prophages (6). The rac prophage contains a gene called *kil*, the product of which causes lethality to the cells. Rho-dependent termination occurs inside the *racR* gene (Figure 1A and Supplementary Figure S3; also see Supplementary Figures S11 and S12 for experimentally determined termination regions) to suppress the downstream *kil* gene expression. We have named this terminator as *t*_rac_. Failure of Rho-dependent termination at the *t*_rac_ causes lethality.

MG1655*rac^+^*strains were at first transformed with the pCL1920 plasmids having WT and different mutant Rho genes, and subsequently a *rho::kan^R^* cassette was transduced (Supplementary Figure S4A). Upon re-streaking the transductants, we observed severe growth defect in the presence of SBS mutants, whereas the PBS mutants did not affect the cell viability significantly (Figure 1B). It was also observed that the transductants obtained in the presence of SBS mutants were not healthy and were few in number (Supplementary Figure S4A). We confirmed the absence of duplication of chromosomal *rho* in all the different transductants, and therefore presence of a WT copy of *rho* is not the cause of viability of the PBS mutants (Supplementary Figure S4B).

Next we measured the termination efficiency of these Rho mutants at *t*_rac_ and at the well-known *t*_R1_ terminators (11). These terminators are fused upstream of the *lacZYA* reporter, and the β-galactosidase activity from this reporter gives the measure of read-through efficiency through the terminators. The termination efficiency of these terminators is inversely proportional to the β-galactosidase activities. As SBS mutants are lethal in the presence of rac prophage (Figure 1B), we performed these assays in MC4100 strain that has lost the rac prophage during propagation (see strain list in Table 1).

SBS mutant, N340S, was observed to have severe termination defects at both the terminators (∼18-fold increase in β-galactosidase activity). The PBS mutants, on the other hand, were severely defective only on *t*_R1_ terminator (11–12-fold increase in activity; Figure 1C and D). The defect caused by PBS mutants on the *t*_rac_ terminator was relatively milder (∼5-fold increase in activity) compared to *t*_R1_*.* This result is consistent with the observation that PBS mutants do not affect the viability of the cells that primarily arises due to severe termination defect at *t*_rac_ (Figure 1B).

These results indicate that defects of PBS mutants are terminator-specific. To check whether this differential effect of PBS mutants is general in nature, we decided to monitor the defects in genome-wide termination patterns of these Rho mutants.

### Differential genome-wide expression profiles for PBS and SBS mutants

We performed microarray analyses of MG1655*Δrho* strain expressing WT and mutant Rho genes from a low copy pCL1920 plasmid and plotted the fold-change in gene expression of different mutants relative to that of the WT (signal intensity with respect to the WT; Figures 1E–H and 2A, Supplementary Figure S5A and S5B). For SBS mutants, MG1655*rac^−^* strains (devoid of rac prophage) were used. We observed the following.

1. Among the genes expressed in the mid-log phase, ∼500–600 genes are affected due to the presence of the SBS mutants, and this is comparable to that observed in the presence of the Rho inhibitor, bicyclomycin (BCM; Supplementary Figure S5A; 6). Majority of the genes affected by the SBS mutants were also affected in the presence of BCM (Supplementary Figure S5C). The number of affected genes is about 5-fold (∼100 genes) lower in case of the PBS mutants. A list of genes that are significantly affected by the SBS mutants but not by the PBS mutants is given in Supplementary Figure S6. The up-regulated genes in the microarray profiles could directly be attributed to the defect in Rho-dependent termination functions, and are also indicative of the presence of Rho-dependent terminators in those operons. However, up-regulation of some of the operons could also be due to activations of the promoters by certain transcription regulators expressed from the Rho-dependent termination defects in other operons. The reasons for down-regulations are likely to be mostly indirect.
2. Consistent with the fact that the viability of MG1655*rac^+^* strains was unaffected in the presence of PBS mutants (Figure 1B), the microarray data also showed that expression of rac prophage genes was not affected by these mutants (Figure 1I and J). Rac prophage genes, however, were up-regulated in the presence of Rho inhibitors, Psu (Supplementary Figure S5D; 18) and BCM (Supplementary Figure S5D). As the microarray profiles of SBS mutants were obtained from an MG1655*rac^−^*, gene expression from the rac prophage was not observed.
3. Most up-regulated genes by the PBS mutants are tabled in Supplementary Figure S7A. Most of these genes or non-coding regions were also up-regulated by the SBS mutants with comparable extent. Notable examples are the cysteine biosynthesis operons, *rho* and many *y* gene operons (unannotated), as well as some of the intragenic non-coding regions. Up-regulation of *rho* is consistent with the fact that its expression is terminated by a Rho-dependent terminator located upstream of this gene that directly binds rho when the latter's concentration is elevated in the cell (24). Up-regulation in some of the non-coding regions is likely from the transcription read-through activities from the neighboring genes (Supplementary Figure S7B).
4. Figure 2A shows the plots of both coding (76 genes) and non-coding genes (24 genes) for which the differential effects of SBS and PBS mutants are maximum. The list of these genes is provided in Supplementary Figure S6. These genes or operons are likely to have Rho-dependent terminators, which are specifically not affected by the PBS mutants. Like rac prophage, other prophage (prophages CP-4-6, Qin, DLP-12, CP-44) genes were also observed to remain unaffected by PBS mutants (Supplementary Figure S6, indicated in color; compare the fold-change numbers). It was interesting to observe that a robust primary RNA binding function of Rho was not essential to execute one of its most important functions like suppression of prophages (6).

Next, we validated some of the microarray data by qRT-PCR. We have randomly chosen three genes. Among them, *gshA* was up-regulated to a comparable level by both N340S and Y80C mutants, while only SBS mutants increased the levels of *yagM* and *ybcK* mRNA (Figure 2B). We performed qRT-PCR using primers corresponding to these genes and observed consistent results with those obtained from microarray experiments (Figure 2C).

The aforementioned data strongly indicated that the tolerance of mutations in the primary RNA binding domain of Rho is not confined to one specific terminator, *t*_rac_, but is a genome-wide phenomenon. Therefore, we concluded that only a subset of Rho-dependent terminators actually requires a stable interaction of RNA (*rut* sites) with the primary RNA-binding domain of Rho.

### *In vitro* defects of PBS mutant of Rho

Next, we tested the differential effects of the PBS (Y80C) and SBS (N340S) Rho mutants in *in vitro* transcription reactions using purified components. We used a DNA template having the *t*_rac_ terminator embedded inside the *racR* region (Supplementary Figure S3A), where transcription was initiated from its natural terminator P_RM_. We used *t*_rac_ as a prototype of the subset of terminators that are not affected by PBS Rho mutants for further *in vitro* and *in vivo* studies. Transcription termination at these terminators was compared with the classical Rho-dependent terminator *t*_R1_, where all the Rho mutants are defective (11). Assays were performed both in the presence and absence of NusG. We have also included few *in vivo* and *in vitro* transcription termination assays using the *ybcK* and *yNM* operons that were shown to have *t*_rac_*-*like terminators in the microarray and qPCR assays (Figure 2B).

From the *in vitro* transcription assays showed in Figure 3A, we observed that the Y80C and N340S mutants were defective for both the terminators in the absence of NusG. However, NusG could partially improve the termination activity of Y80C only on the *t*_rac_ terminator (Figure 3A, left panel). NusG does not have much effect on the SBS mutants. We also observed that in the presence of WT Rho, NusG exerts significantly greater effect (more pronounced early termination) on the *t*_rac_ terminator than on *t*_R1*.*_ Another *t*_rac_-like terminator, *t*_yag_, also exhibited similar NusG dependence (Supplementary Figure S11, third lane of *t*_yag_ panel). The partial restoration of the termination activity of Y80C by NusG on the *t*_rac_ terminator and the strong effect of NusG on this particular terminator led us to hypothesize that *in vivo* NusG might suppress the defects of primary RNA binding function on the *t*_rac_-like terminators (Figure 1B).

### Effect of NusG on the *t*_rac_-like terminators *in vivo*

Earlier *in vitro* studies indicated that NusG improves the EC dissociation and does not have significant effect on Rho–RNA interactions (12,15). The aforementioned *in vitro* data, however, hinted a modulation of primary RNA binding functions of Rho by NusG.

To test this, we first monitored the *in vivo* NusG dependence of the terminators, *t*_R1_*, trpt′, t*_rac_, and also another *t*_rac_*-*like terminator*, t*_ybcK_ (untranslated region of *ybcK*; Supplementary Figure S8a), which displayed differential responses to the PBS and SBS mutants (Figures 1 and 2B and C). We measured this dependence quantitatively by measuring the β-galactosidase activities of the *lacZ* reporters fused downstream of these terminators (Figure 3B) in the presence of WT and three NusG-CTD mutants, G146D, V160N and L158Q. The effects of the NusG mutants on the terminator (residing in the untranslated region) read-through activities resulting into high-level transcription of the *ybcK* were measured by RT-PCR (Figure 3E) using gene-specific primers (Supplementary Figure S8A). These NusG mutants are defective in binding to Rho (Figure 3C; 12). These mutants were expressed from plasmids. The strain, MC4100*rac^−^*, was deleted for the chromosomal *nusG* and also for rac prophage. The latter deletion also allows the strain to grow in the presence of NusG mutants defective for Rho-binding. In this assay, values of the β-galactosidase activity as well as the amount of RT-PCR products are proportional to the extent of termination defects; stronger defects make terminators more NusG-dependent.

We observed that the β-galactosidase activity was enhanced by 30–40-fold in the presence of NusG mutants when the *t*_rac_*–lacZ* fusion was used, whereas mere increase of 2–2.5-fold was seen for *t*_R1_–*lacZ* and *tr1–trpt′*–*lacZ* fusions (Figure 3D; compare the values for WT and the mutants and also note the break in the scale). In case of *t*_ybcK_, the intensity of RT-PCR product was observed to increase by ∼3–3.5-fold in the presence of NusG mutants (right panel of Figure 3E), whereas the effect was marginal in case of *t*_R1_*–lacZ* fusion (left panel of Figure 3E). In the latter case, the RT-PCR primers correspond to a region inside the *lacZ* gene (see schematics of Figure 3B).

These results strongly suggest that the *t*_rac_ as well as *t*_ybc_ terminators are highly dependent on NusG for their termination function. It is likely that in *t*_rac_-like terminators, the weaker interactions at the PBSs are compensated by the additional free energy available from the Rho–NusG interactions.

If *t*_rac_*-*like terminators are so much NusG-dependent, it is possible that NusG mutants may specifically produce synthetic lethality with the Rho PBS mutants. We constructed two MC4100 strains having G146D and L158Q mutations in the chromosomal *nusG.* These strains also lacked the rac prophage, so that the *nusG* mutants could be moved to their chromosome readily, and subsequently could also be transformed by plasmid expressing the SBS mutant, N340S. We at first transformed these two strains with pCl1920 plasmids having either WT or Y80C (PBS mutant) or N340S (SBS mutant) mutants of *rho.* Finally, the chromosomal *rho* was deleted by P1 transduction and the growth of the transductants upon re-streaking on LB plates was monitored. Figure 3F shows that the combination of both G146D and L158Q NusG specifically with the PBS mutant, Y80C, was lethal. This synthetic lethality was not so pronounced with the SBS mutant, N340S. However, in the latter case, the growth was slower than that of WT.

These results strongly suggest that NusG specifically helps the PBS mutants *in vivo*, and further reinforced the proposition that Rho recruitment steps of the *t*_rac_-like terminators are NusG-assisted.

### Comparison between the rut sites of *t*_rac_-like and *t*_R1_-like terminators

The above results indicate that depending on the mode of Rho recruitment, the Rho-dependent terminators could be divided into two categories: *t*_R1_-like and *t*_rac_*-*like terminators. We compared the sequence properties of the rut regions of these terminators (Supplementary Figures S9 and S10 and Supplementary Figures S8A and B for the sequence of the terminators in *ybcK* and *yagNM* operons, respectively) and also calculated the distances of their respective termination zones from the rut sites from *in vitro* as well as *in vivo* termination assays (Supplementary Figures S11 and S12). As the termination zones of the *t*_rac_ terminator are inside the *racR* (Supplementary Figure S3), we assumed that the Rho-loading site(s) would be in the preceding untranslated region (+1 to +210). From the sequence analyses (Supplementary Figure S9), we observed that the segments of region +60 to +120 have the lowest propensity of forming secondary structures (ΔG = −2.3 kCal/mole) and high C/G ratio. Sequence composition of the adjacent region, +1 to +60, also has a higher C/G ratio like other classical terminators (Supplementary Figure S9). Hence, according to the definition of a Rho-binding site (1), these region(s) of *t*_rac_ terminator match the properties of bonafide *rut* site(s), and these sites are expected to offer optimal interactions to Rho, in a similar way as the *t*_R1_*-*like terminators. This assumption is also consistent with the fact that, *in vitro*, Rho terminates efficiently on *t*_rac_ in the absence of NusG (Figure 3A), and therefore this terminator is likely not to have significant defect in Rho recognition.

In a similar manner, assuming the untranslated regions of *ybcK* and *yagNM* operons contain the *rut* site, we analyzed these regions for their propensity to form secondary structures and calculated the C/G ratio (Supplementary Figure S10). We observed that 61–120 nt and 41–87 nt of the untranslated regions of *ybcK* and *yagNM* operons, respectively, have low free energy of secondary structure formation and high C/G ratio.

Upon comparison of the distance between the Rho-loading regions and the termination zones of different terminators (Supplementary Figures S11A and B; compare the termination zones of *in vitro* transcription assays), we noticed that the mean distances of the termination zones for *t*_rac_ and *t*_yag_ (*t*_rac_*-*like terminators) are unusually long compared to the *t*_R1_*-*like terminators (Supplementary Figure S11; ∼385–425 nt versus 70–145 nt of the classical terminators). Using *in vivo* termination assays, we have also confirmed that for *t*_rac_ the termination region is indeed located inside the *racR* (Supplementary Figure S12). These observations can be interpreted as much longer time requirements of Rho on these two terminators to dislodge the EC. Assuming that the translocation speed of Rho remains comparable on different RNA sequences, the following two parameters should determine the aforementioned distance as well as the time taken to dissociate the EC: the time taken to form a translocase-competent Rho at the *rut* site and the time taken to dislodge the EC. The latter parameter depends on the nature of the EC (20), and hence, following the Rho-loading, isomerization step(s) of forming a translocase-competent Rho is likely to regulate the rate of the whole process (see Figure 5). This theoretical framework predicts that the rate(s) of isomerization step leading to the formation of a translocase-competent form at *t*_rac_-like terminators is slower as compared to that of the *t*_R1_-like terminators. In this context, we also propose that the distance between the *rut* site and the termination zone can be used to determine the strength of a Rho-dependent terminator.

**Figure 4.**
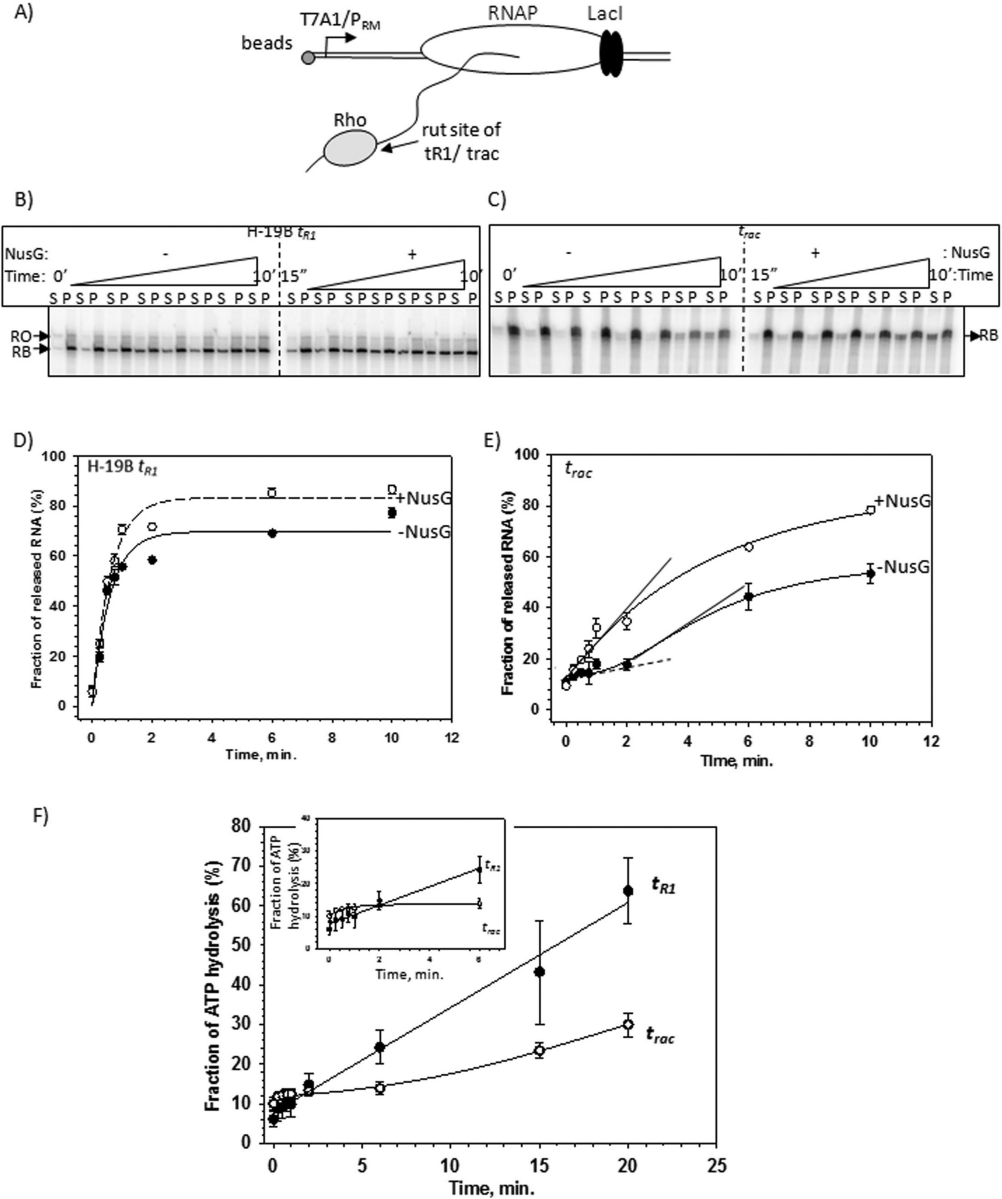
(**A**) Schematic showing the design of the formation of stalled elongation complexes (RB) downstream of the rut sites of either H-19B *t*_R1_ or *t*_rac_ terminators using lac repressor. Different components of the quaternary complex have been indicated. The length of the nascent RNA is 374 nt and 435 nt for RBs with *t*_R1_ and *t*_rac_ terminators, respectively. (**B**) and (**C**) Autoradiogram showing the Rho-induced RNA release kinetics from the RBs formed downstream of indicated terminator sequences both in the absence and presence of NusG. ‘S’ denotes half of the supernatant and ‘P’ denotes rest of the sample. RB denotes the RNA corresponding to the position of the stalled EC and RO indicates the run-off product of this template. (**D**) and (**E**) Fractions of released RNA in the above experiments were plotted against time for the indicated RB complexes formed downstream of the two terminators. Plots obtained in the absence (-•-) and presence of (-o-) NusG are indicated. The experimental data points were fitted either to an exponential rise form or to a logistic function as described in the Materials and Methods section. The delay in the initiation of RNA release for *t*_rac_ has been indicated by a flat slope drawn as a dashed line. Rates of RNA release of both the curves are indicated by the slopes drawn as solid lines. Note that the rate estimated for −NusG curve is from the incremental phase following the lag phase of the curve. (**F**) Fraction of ATP hydrolyzed by Rho was plotted against time. These reactions were induced by the nascent RNA from the stalled ECs (as described in Figure 4A) formed downstream of the indicated terminators. A significant lag to initiate the ATP hydrolysis was observed at the *t*_rac_ terminator. An amplified version of this lag is shown in the inset.

**Figure 5.**
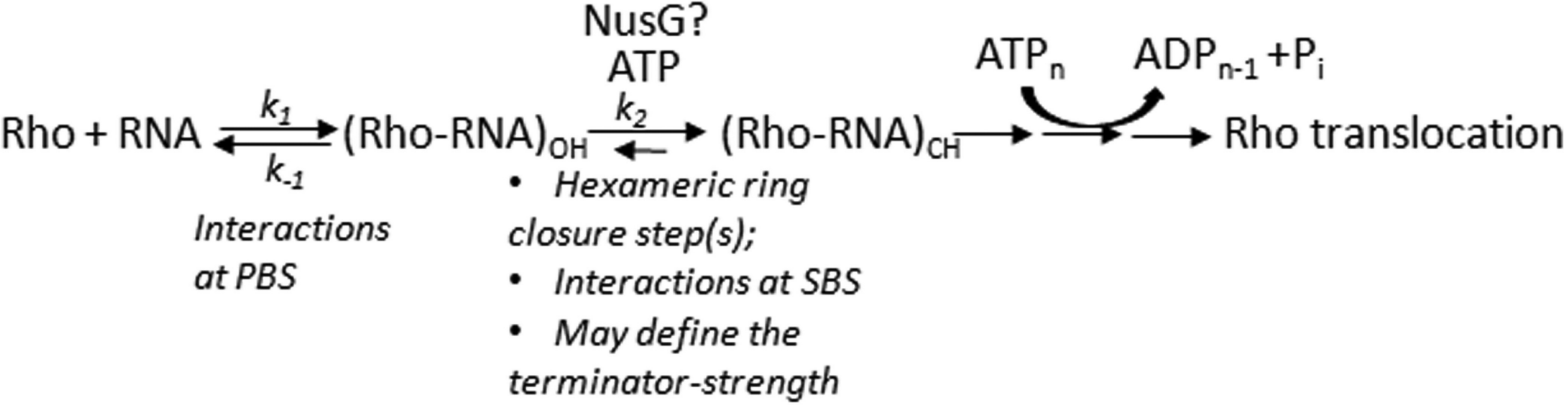
A kinetic scheme showing different steps in the Rho-recruitment process. OH and CH denote the ‘open’ and ‘close’ hexamer states of Rho as described in the two crystal structures (25,26). It is likely that the major isomerization step as well as the rate-limiting step involves the OH to CH conversion, which is induced by threading of the RNA into the SBS and by the binding of ATP. Rho translocation events denoted as ‘→→→’ are accompanied by sequential ATP hydrolysis steps. The rate constants defining the binding steps are also indicated. Proposed involvement of NusG at the isomerization step is shown.

### Probing the isomerization event from the RNA release and ATP hydrolysis kinetics at the *t*_R1_ and *t*_rac_ terminators

The aforementioned isomerization event is expected to be manifested as a ‘lag’ in either Rho-induced RNA release kinetics or nascent RNA induced ATP-hydrolysis kinetics. Slower the isomerization rate, longer will be the period of lag. We probed this lag period by measuring the Rho-induced RNA release kinetics (Figure 4B–E) from stalled ECs formed downstream of either *t*_rac_ or *t*_R1_ terminators (Figure 4A). The nascent RNAs attached to these two stalled ECs were also used to induce ATP hydrolysis of Rho (Figure 4F).

At first, stalled ECs (RB complexes) were formed at the *lac* operator site using lac repressor as a road block on immobilized templates (schematic in Figure 4A). Rho, in the presence of 1 mM ATP, was added to the RBs having the nascent RNAs with either *t*_R1_ or *t*_rac_ terminator sequences. The amount of released RNA (terminated RNA) in the supernatant (S) was followed over a time period (Figure 4B–E). We observed the following: (i) on the *t*_R1_ terminator, the RNA release kinetics was fast and did not show any lag of initiation of RNA release within the time window that was used (0.25–10 min). NusG did not have appreciable effect on the rate of RNA release, but improved the overall efficiency of the process modestly (Figure 4D; compare the ‘+’ NusG and ‘−’ NusG curves); (ii) on the *t*_rac_ terminator, in the absence of NusG, the RNA release was observed to start after a significant lag-time (Figure 4E; slope denoted by a dashed line). This lag disappeared in the presence of NusG. However, NusG, like in case of *t*_R1_ terminator, also improved the termination efficiency of *t*_rac_ but had a modest effect on the rate of RNA release (Figure 4E; compare the slopes indicated as solid lines).

Next we followed the nascent RNA induced ATP-hydrolysis rate of Rho using the same RBs described in Figure 4A (Figure 4F). Similar to the RNA release kinetics, ATP hydrolysis at the *t*_rac_ terminator also exhibited a significant delay in starting the hydrolysis process, whereas the initiation of the process at the *t*_R1_ terminator was fairly instantaneous.

This *t*_rac_-specific lag observed in both the events is consistent with the slow isomerization process at the Rho–mRNA complex formation steps that we predicted from the unusually long distances between the rut site and the termination zone of this terminator (Supplementary Figure S11). These results also indicated that NusG accelerates the isomerization process on the *t_rac_-*like terminators.

## DISCUSSION

*In vitro*, *rut* site-dependent Rho recruitment to the EC was studied using a few strong terminators, such as *λt*_R1_, *trpt′*, etc. (Supplementary Figure S1; 1–2,27). More recent studies have questioned this RNA-dependent recruitment model of Rho (Supplementary Figure S1; 13,14). Here, we have attempted to understand the mechanism of *in vivo* recruitment of Rho to the EC. We concluded that the majority of the terminators do not require robust primary RNA binding functions *in vivo* and follow a NusG-assisted RNA-dependent recruitment pathway based on the following evidences. (i) Growth assays and genome-wide expression analyses showed that Rho PBS mutants, unlike SBS mutants, neither induced growth defects of the cells nor induced termination defects in a large number of genes. These unaffected terminators include those present in rac and other prophages, where suppression of their induction is one of the essential functions of Rho (Figures 1 and 2, Supplementary Figures S5, S6 and S7). (ii) *t*_rac_, *t*_ybck_ and *t*_yag_, examples of this class of terminators, are highly NusG-dependent *in vivo* and *in vitro* (Figures 3 and Supplementary Figure S11). (iii) NusG mutants defective for Rho-binding caused synthetic lethality only with PBS Rho mutants (Figure 3). (iv) The RNA release as well as ATP hydrolysis at *t*_rac_ terminator is accompanied by a long lag period before the initiation of the events, which indicates slow isomerization steps during the Rho-loading steps. The presence of NusG accelerates this slow isomerization process at *t*_rac_*-*like terminators (Figures 4 and 5). Finally, we propose that the terminator strength could be defined by the length of the ‘time-lag’ required to form the translocase competent Rho at each terminator.

Rho binding site(s) on the mRNA are ambiguous in nature, defined by its C-richness and lack of secondary structures (2). And hence, at the weaker interaction sites, Rho–NusG interaction will provide an additional free energy for Rho–RNA complex formation. As NusG is always bound to the ECs, there is a possibility that Rho–mRNA and Rho–NusG interaction occurs simultaneously or within a very short time window. However, the effect of NusG at the recruitment stage is not measurable at the sites of strong Rho–RNA interactions, as the additional stabilization forces are not necessary in these cases.

Based on the two forms of crystal structures of Rho, open hexamer (OH) and closed hexamer (CH) (25,26), and consecutive interactions of RNA at its PBS and SBS, we defined the Rho-loading as a multi-step process, which eventually leads to the formation of a translocation competent form (Figure 5). The massive conformational changes involving hexameric ring closure and activation of SBS-RNA interaction induced ATP hydrolysis should be manifested as slow isomerization step(s). Our results indicate that the rate of this isomerization depends on the nature of the terminators (*t*_R1_-like versus *t*_rac_-like) and is likely to be dictated by the RNA sequences in and around the *rut* sites. Stability of Rho–RNA interaction will depend mostly on isomerization rate constant (*k_2_*). As the formation of the translocase-competent form is irreversible in nature, a higher value of *k_2_* will drive the equilibrium toward forming this state by overcoming the reversible Rho–RNA binding step(s). We suggest that the terminator strength will be dependent on the values of this rate constant. The value of *k_2_* will be higher in case of strong terminators. We also propose that Rho–NusG interaction increases the *k_2_* at the weak terminators and thereby overcomes the defects in primary RNA binding function. This could be a general strategy utilized by Rho when it encounters weaker sites on the mRNA. It should be noted that the putative NusG binding region on Rho is quite distal to the secondary RNA binding channel of Rho (12), so NusG effect on the isomerization step is likely to be allosteric in nature.

Because in the *in vitro* transcription reactions NusG induces early termination and increases the speed of RNA release, it was thought to function at the RNA release step of Rho-dependent termination (10–12). If NusG accelerates the aforementioned isomerization step(s), it will reduce the translocation time of Rho to reach the EC, and hence will cause early termination. A recent ChIP-seq study (13) concluded that terminators with ‘poor’ *rut* site are more dependent on NusG. According to our methodology to define the terminator strength (Supplementary Figure S11), the unstable interactions between SBS and the sub-optimal *rut* sequences are likely to slow down the isomerization steps (having lower *k_2_*). NusG suppresses this defect and these terminators exhibit enhanced dependence on NusG.

Optimal function of the *t*_rac_-like terminators will also depend on the availability of free NusG-CTD to Rho. It is likely that the ribosome-bound S10-NusG interaction (28) will regulate the strength of these NusG-dependent terminators. Finally, NusG-CTD brings Rho closer to the RNAP and, therefore, a possibility of Rho–RNAP interaction during the Rho-recruitment stage, at least on the *t*_rac_*-*like terminators, cannot be ignored. However, this Rho–RNAP interaction is not likely to dislodge the EC because Rho translocation along the mRNA *a priori* to the RNAP dissociation is mandatory (Figure 1). This ‘idle’ Rho–RNAP interaction was envisioned earlier (13,14) and very recently has been observed in the *in vivo* cross-linking experiments (29).

## SUPPLEMENTARY DATA

Supplementary Data are available at NAR Online.

SUPPLEMENTARY DATA
