# Supplementary material for: Redundancy of primary RNA-binding functions of the bacterial transcription terminator Rho
Source: Nucleic Acids Res. 2014 Jul 31;42(15):9677–90. doi: 10.1093/nar/gku690 (PMC4150792; doi:10.1093/nar/gku690)
Supplement: SUPPLEMENTARY DATA [file supp_42_15_9677__index.html]

Redundancy of primary RNA-binding functions of the bacterial transcription terminator Rho — SUPPLEMENTARY DATA 

# Redundancy of primary RNA-binding functions of the bacterial transcription terminator Rho

## SUPPLEMENTARY DATA

**Files in this Data Supplement:**

- SUPPLEMENTARY DATA
