# Supplementary material for: Redundancy of primary RNA-binding functions of the bacterial transcription terminator Rho
Source: Nucleic Acids Res. 2014 Jul 31;42(15):9677–90. doi: 10.1093/nar/gku690 (PMC4150792; doi:10.1093/nar/gku690)
Supplement: SUPPLEMENTARY DATA [file supp_gku690_nar-01163-r-2014-File007.pdf]

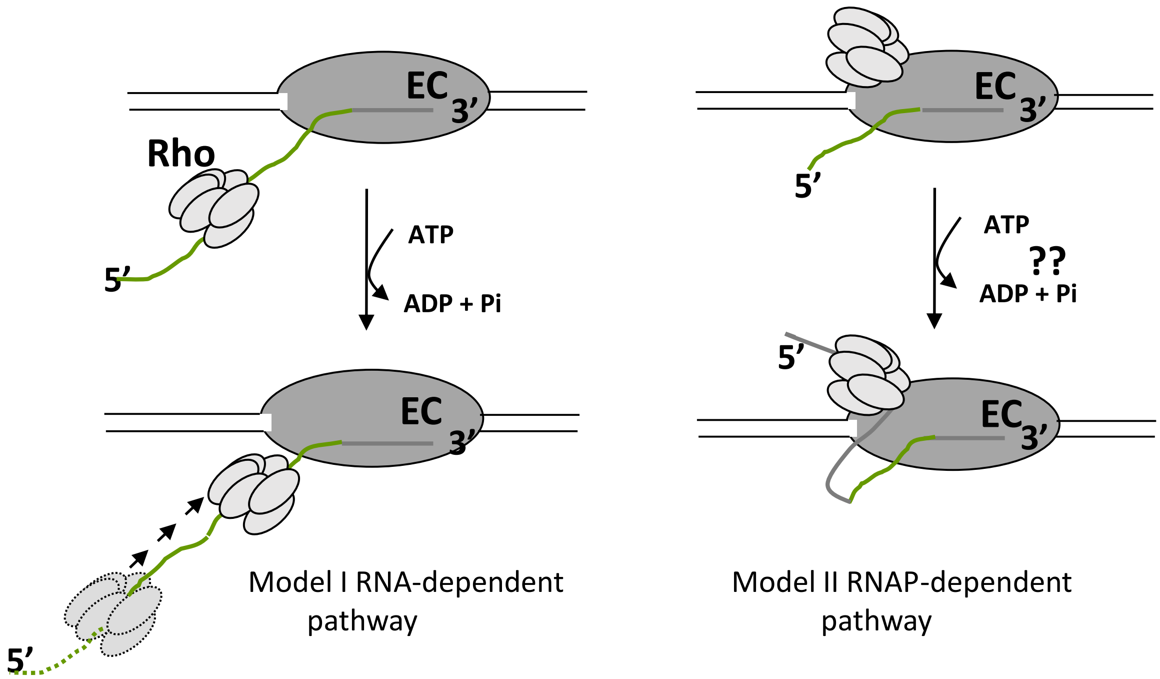


**Figure S1:**

Model I: Rho is recruited to the EC by binding to the nascent RNA via the *rut* site, translocates along the RNA, and eventually catches up the EC and dislodges it. This model predicts that RNAP-elongation and Rho-translocation have to be kinetically coupled[1].

Model II: Rho forms complex with EC prior to loading onto the RNA following which Rho gets activated to drag the mRNA through itself, and termination occurs by allosteric modification of the EC. Kinetic coupling as well as translocase activity of Rho may not be required in this model. Also *rut* site mediated recruitment of Rho is not necessary[13,14].

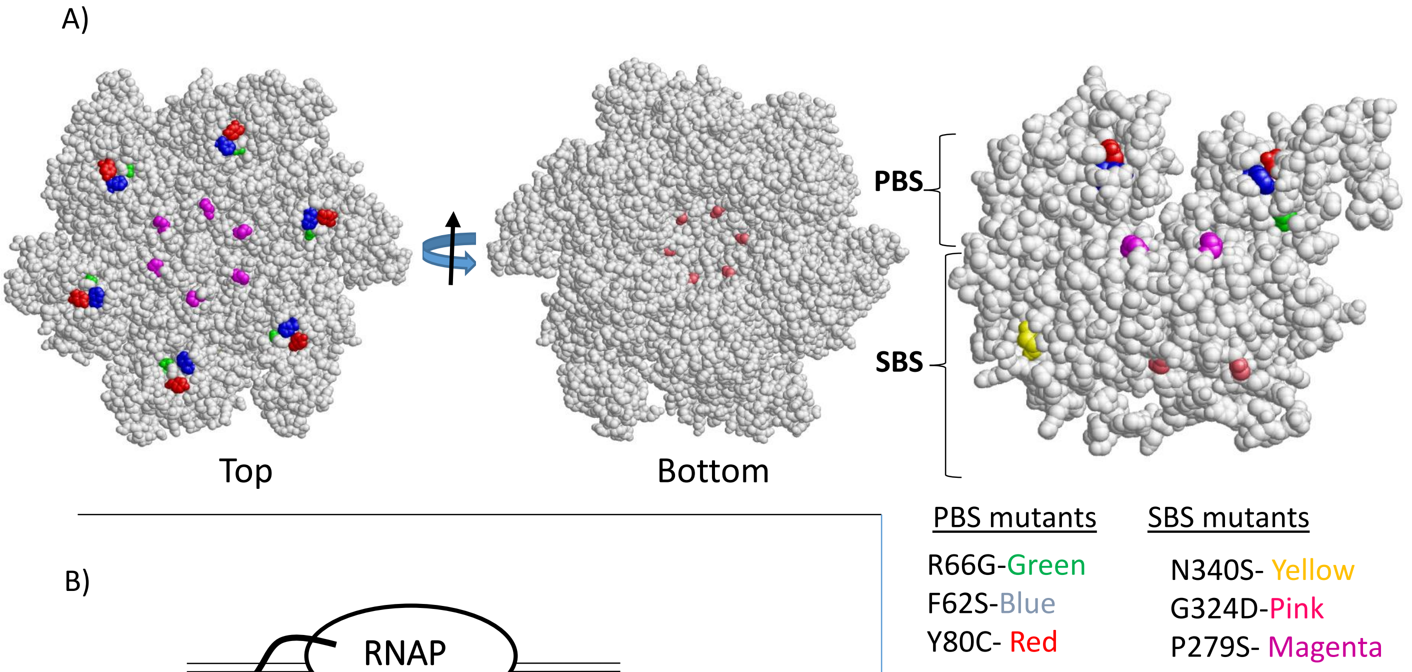


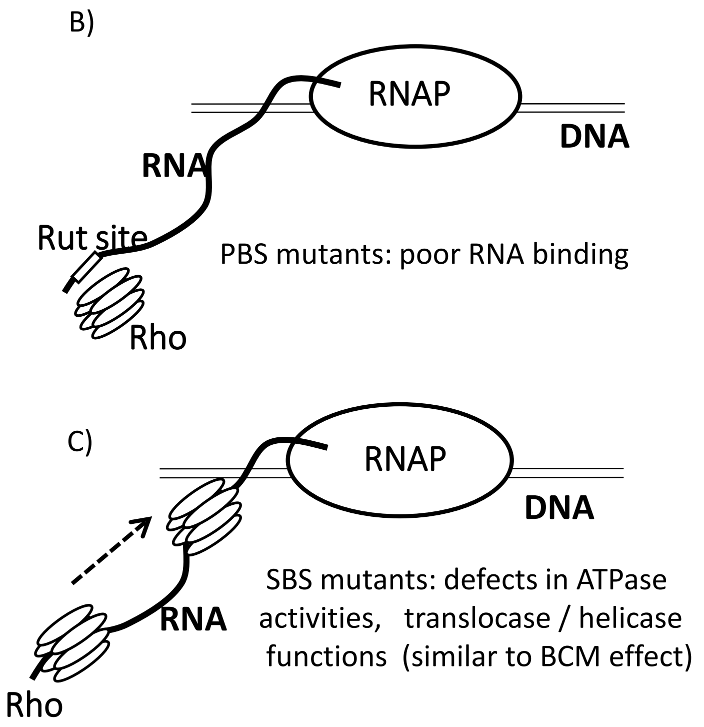


D)

**Binding affinity of the PBS Mutants**

| Rho | $K_d$ |
|---|---|
| Wild type | 10±0.9 $(dC)_{34}$ nM[11] |
| Y80C | >3000 nM[11] (polyC) |
| Wild Type | 5±1.6 $(rC)_{10}$ μM[16] |
| F62S | Too low, no binding[16] |

Figure S2: A) Locations of different point mutations in Rho. Mutations in primary and secondary RNA binding regions are indicated. Top view shows the primary RNA binding site, while the bottom view shows the RNA exit channel and secondary RNA binding sites. B) and C) Functional defects of PBS and SBS mutants explained through cartoons. D) Experimentally determined binding constants of WT and PBS Rho mutants for DNA and RNA molecules those are indicated in the parenthesis.

*Early region of rac prophage containing the racR/$t_{rac}$ terminator sequences cloned before a lacZ reporter*

RS83

CCATAAACTG CCAGGAATTG GGGATCG***GAA TTC***GCTTCAC TGACATATTC TGCGAACAAC ATGCCGAACG
TCGTAAATAT GACCAGTCAA TATCAGGACGAAG TTCTTCGCAC AGAACCTCAC CTCTTGTTGC ACGTTCAATT }$P_{RM}$
GCTGGACATC TCTCGGCAGG CAA***TTGACG***T ACCCCTTTGA TCCATTGATT ***TACGCTT***GGA GGTGATACAC
CTAAAAGCCT AGCCATTGCT GATTGCCCAC CGACAACAGC ACAAGCTTGC TTGAATGAAT AGTTCTCTTT ***Untranslated***
TTTCATCGAA TGAACTCCAA AAACACACAG AAATATTAGG CGACGCCTAA CGCAATTGTC AATAGGCTGT ***region***
GCCTAATGCA GTAAGGGTAG GGATTGCCTA ATGTAATGCG CATAGGAGAA TATTAAGCA ***ATG***CTTAGTG
GTAAAGACTT AGGCCGAGCG ATAGAGCAGG CCATTAACAAA AAAATCGCA TCGGGATCCG TCAAATCAAA
GGCGGAGGTC GCACGCCACT TTAAAGTCCA ACCACCATCA ATTTATGACT GGATTAAGAA AGGCTCTATA
AGTAAAGATA AACTTCCAGA ATTATGGCGT TTCTTTTCTG ATGTTGTTGG TCCAGAGCAT TGGGGGCTTA ***racR***
ACGAATACCC CATACCAACC CCCACCAATT CAGATACAAA AAGTGAACTT TTAGATATAA ACAACCTTTA
RS955
TCAAGCAGCC TCTGATGAAA TAAGAGCGAT TGTAGCTTTC CTGTTATCTG GAAATGCTAC AGAACCAGAT
TGGGTTGACC ACGATGTTCG CGCCTACATA GCAGCGATGG AAATGAAAGT GGGTAAGTAT CTGAAAGCTC
RS956
TTGAATCTGA ACGGAAAAGC CAGAACATCA CAAAAACTGG AACTTAA
ACTTATATGG TCTGACGGAA AACTCCTGGA TTCCGTTATT TAACCCCCCC ATCACTTTCT GCTGTCGCCA
TCACCTATTA GGTTACGCTC AAAACATTAG GCATAGCCTA TTGACAATCA ATTAGGCATT ACCTATAGTT ***Intragenic***
CCAGCATACC ACCCACCCCG CCCCACAGAA CGCCGGGCAA TACTTCGAGT TACCAGGCAG TGGTAAGGGG ***region***
TTAAGTAGCC AGCCCGAGGC GTATGAACAT GACGGCGGGA TTCAAATTTT GCAGTGCAGC AGTTAGTTCC
RS845
GCCACCCGGCGTTAAGGGGA*GAGATAAG**GGATCC***
TAACTAACTAGCGATCCCGACTCACTATAGAGGGACAAACTCAAGGTCATTCGCAAGAGTGGCCTTTATGATTGACC
TTCTTCCGGTTAATACGACCGGGATCGAGATCCTAGGTAGGTAGGGGCGCGGCATTTTAACTTTCTTTATCACACAG ***LacZ***
GA AACAGCTATG ACCATGATTA CGGATTCACTGGCCGTCGTTTTACAACGTCGTGACTGGGAAAAC
RS-RK1

***In vitro termination region***

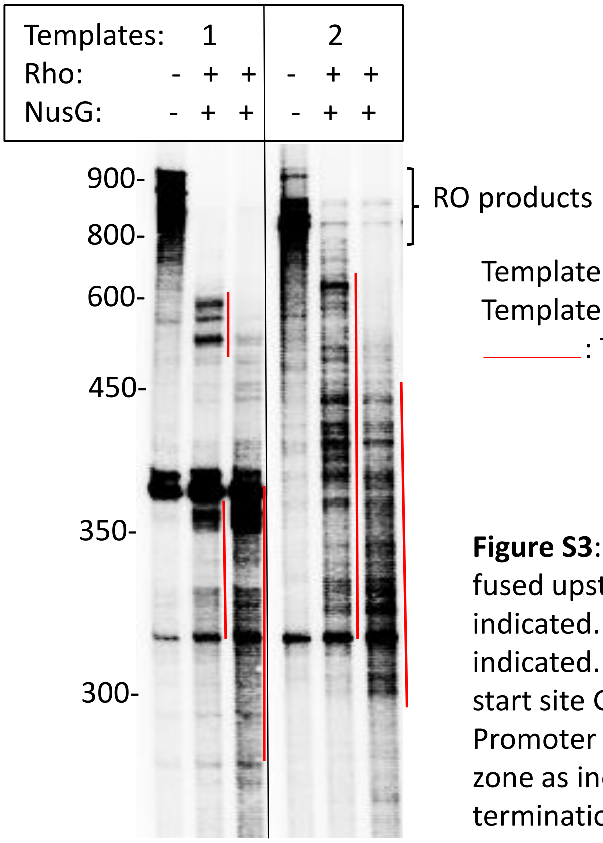


Template 1 made from oligo pair RS83/RK-1
Template 2 made from oligo pair RS83/RS845.
______ : Termination regions

**Figure S3**: Sequence of the $P_{RM}$-$t_{rac}$-*racR* region of rac prophage fused upstream of a *lacZ* cassette. Different regions are indicated. Primers used for making the DNA templates are also indicated. In the *in vitro* transcription assays the transcription start site G, shown as a small arrow, is utilized by the RNAP. Promoter is shown in yellow, *racR* in blue highlights. Termination zone as indicated was obtained from *in vitro* and *in vivo* termination assays (see adjacent autoradiogram and figure S8).

A)

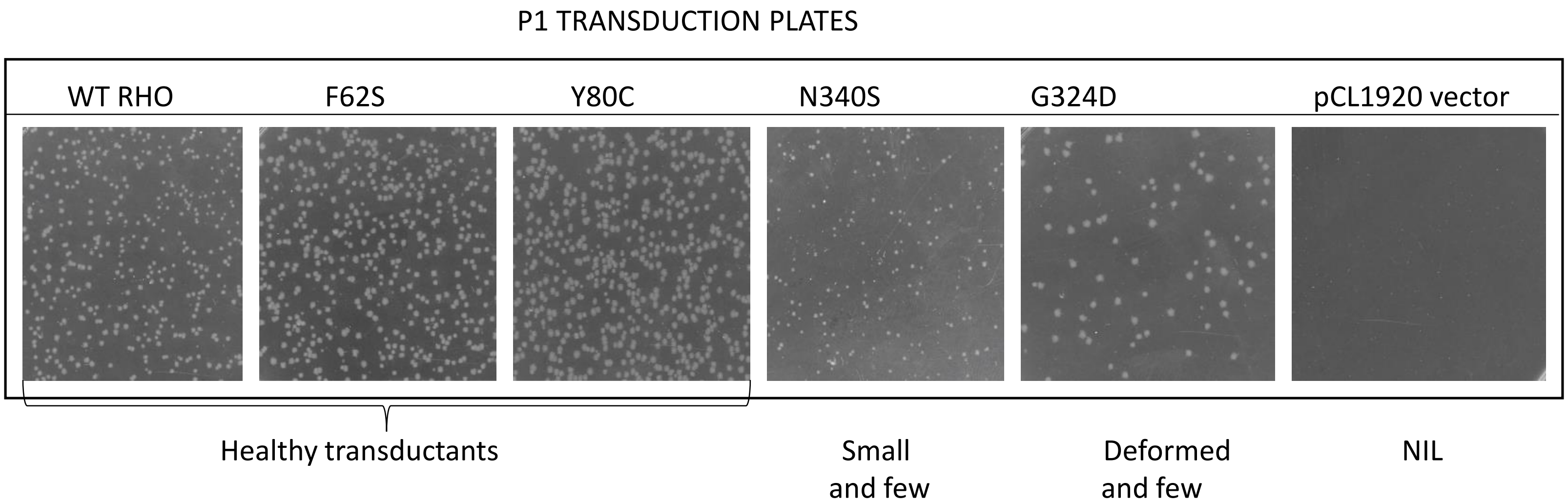


**Figure S4A**: *MG1655 rac⁺* strains were at first transformed with pCL1920 plasmids expressing WT and different derivatives of *rho* mutants as indicated on the top of the pictures. Resultant strains were P1 transduced with a *rho:kan*$^R$ cassette to delete chromosomal *rho*. The pictures showed the number and nature of the transductants obtained in the presence of WT and different rho derivatives as indicated. In cases of WT and the two PBS mutants, Y80C and F62S, transduction efficiency was high and the transductants were healthy-looking. In case of N340S, transductants were small, whereas these were deformed when G324D was present. The transduction efficiency was also poor for these two SBS mutants. There were no transductants in the presence of empty vector. In figure 1B, these transductants were re-streaked on LB plates and incubated overnight at 37$^o$C.

B)

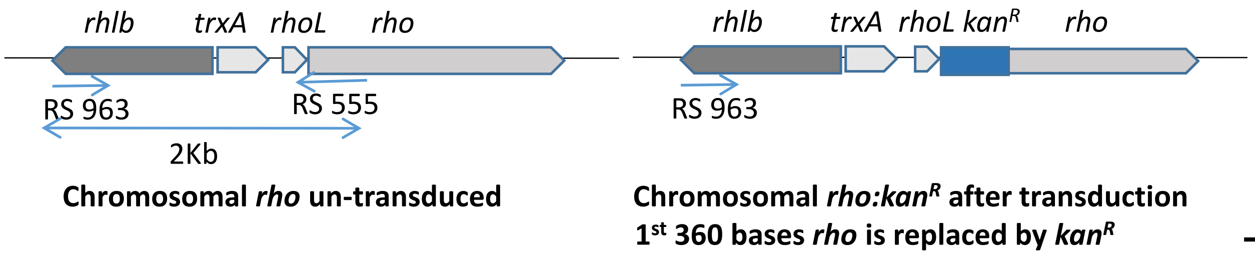


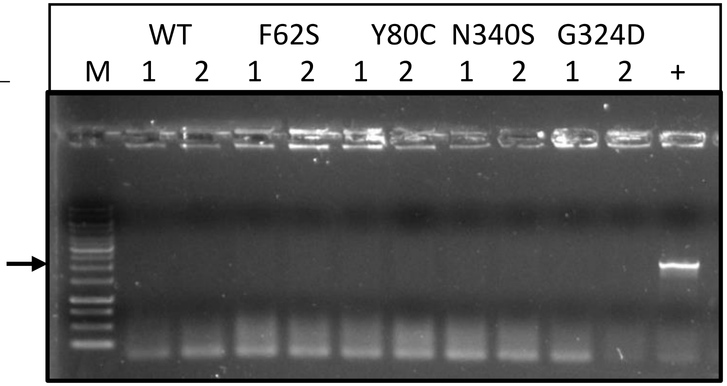


**Figure 4B**: RS963 and RS555 primers were designed such a way that they will produce ~2kB PCR-product only if an intact *rho* is present in the chromosome, and thereby will detect the occurrence of the duplication of *rho.* This primer pair will not produce any PCR product neither from *kan*$^R$ *:rho* nor from the pCL1920 plasmids expressing WT or mutant *rho*s, because in each case either N-terminal of rho or *rhlB* is absent. WT MG1655 strain is used as the "+" control, whereas strains transformed with empty vector were used as a "-" control. Absence of the 2kB PCR product indicates absence of rho-duplication. Two transductants (1, 2) for each of the *rho* mutants were tested.

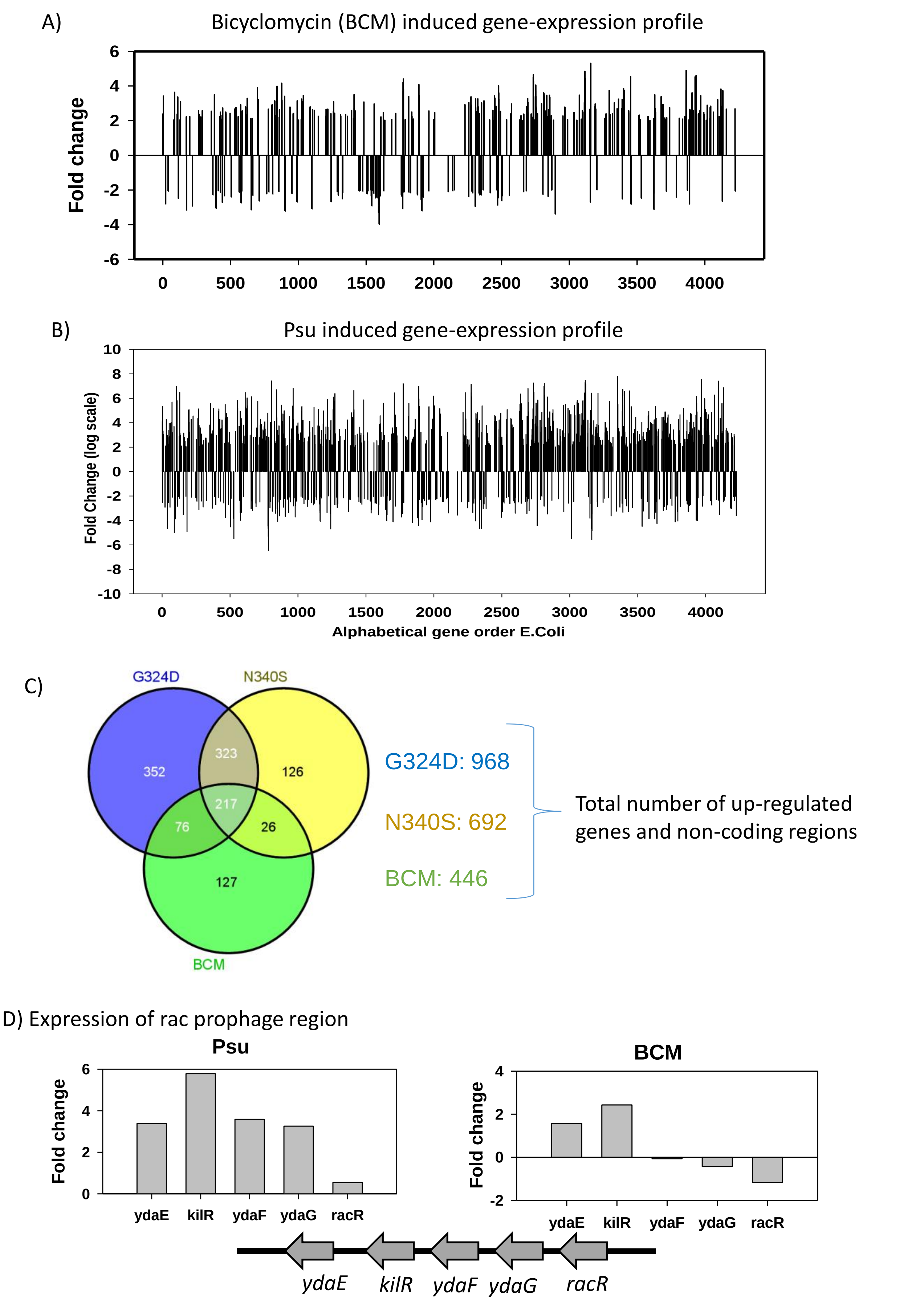


Figure S5. Microarray profiles of genome wide expression patterns of MG1655 treated with A) BCM and B) due to expression of Psu. Psu is expressed from a plasmid and has a drastic effect compared to SBS mutants or BCM. C) Venn diagram showing the overlaps between up-regulated genes of Rho mutants and those obtained after BCM treatment. This is generated using VENNY online tools from http://bioinfogp.cnb.csic.es/ tools / venny/ index.html. D)Expression profiles of the rac prophage genes in the presence of these two Rho-inhibitors. Expression of *kilR* is up-regulated when Rho is inhibited by Psu and BCM.

## Expression of GENES those are less affected by PBS mutants

| Genes | Fold change in gene expression level (Grouped values) | | |
|---|---|---|---|
| | PBS mutants | SBS mutants | |
| aaeA | 0.8560 | 3.7106 | |
| aaeB | 1.9583 | 3.7579 | |
| aaeX | 0.6806 | 4.5353 | |
| allB | 0.2170 | 3.0600 | |
| aqpZ | -0.1951 | 3.8572 | |
| aslB | 1.2368 | 3.5999 | |
| bcsA | 0.2331 | 3.3926 | |
| cadA | 2.2939 | 5.4593 | |
| cadB | 1.2461 | 3.1624 | |
| caiC | 0.3784 | 3.1889 | |
| caiD | 0.2293 | 3.4299 | |
| cbrA | 1.3545 | 3.2240 | |
| cbrB | 0.8678 | 3.1717 | |
| ecpD | 1.0164 | 3.3764 | |
| etp | 2.7829 | 5.0305 | |
| eutD | 1.8017 | 3.3614 | |
| eutG | 0.5982 | 3.3481 | |
| flu | 1.8894 | 3.4883 | |
| fryC | 1.9004 | 3.0495 | |
| gfcC | 2.9529 | 6.0432 | |
| mdtA | 0.8001 | 3.2401 | |
| mutL | 2.0040 | 3.5415 | |
| phoH | 1.8294 | 3.3983 | |
| pitB | 7.6934e-3 | 3.0954 | |
| rhsB | 1.2866 | 3.1663 | |
| sfmH | -0.3877 | 3.1238 | |
| sgcX | 3.6982 | 3.5332 | |
| thiC | 1.5139 | 4.8160 | |
| thiE | 1.6363 | 4.2424 | |
| thiF | 1.6704 | 4.0786 | |
| thiG | 0.8281 | 3.8829 | |
| thiH | 0.8979 | 3.5300 | |
| thiS | 0.9938 | 3.9311 | |
| uhpT | 2.3552 | 4.4967 | |
| **yagK** | **1.8767** | **4.1309** | **CP4-6** |
| **yagL** | **1.1385** | **4.7311** | **CP4-6** |
| **yagM** | **1.4124** | **3.2965** | **CP4-6** |
| **ybcK** | **0.1373** | **4.4540** | **DLP12** |
| **ybcL** | **-0.0450** | **3.7694** | **DLP12** |
| **ybcN** | **1.4722** | **3.7652** | **DLP12** |
| ybeF | 0.8198 | 3.1497 | |
| ybfB | 0.5121 | 3.2270 | |
| ybfD | 1.1082 | 4.3139 | |
| ybfQ | 0.9176 | 5.2946 | |
| yciW | 3.6497 | 3.1442 | |
| ydcC | 0.8906 | 4.4863 | |
| ydeO | 0.3647 | 3.1981 | |
| ydeP | 1.8224 | 3.2871 | |
| ydeQ | 2.1027 | 4.8034 | |
| ydeS | 1.1530 | 3.8827 | |
| **ydfU** | **1.0241** | **3.2317** | **Qin** |
| ydhS | 2.6417 | 7.0625 | |
| ydiO | 0.1592 | 3.1277 | |
| **yeeR** | **2.7713** | **3.8542** | **CP-44** |
| **yeeS** | **2.7728** | **3.2037** | **CP-44** |
| **yeeU** | **1.1379** | **4.0239** | **CP-44** |
| yegR | -0.0215 | 6.0768 | |
| yfbN | 1.0878 | 4.2325 | |
| yfhR | 2.6596 | 4.3419 | |
| ygiZ | 1.0328 | 3.6525 | |
| yhcA | 0.6425 | 3.3130 | |
| yhhH | 1.6212 | 3.1063 | |
| yhhI | 1.8361 | 4.3626 | |
| yhiD | -0.2105 | 3.0049 | |
| yhjH | 1.9629 | 3.5160 | |
| yibV | 0.7608 | 5.0041 | |

| Genes | Fold change in gene expression level | |
|---|---|---|
| | PBS mutants | SBS mutants |
| yjfJ | 0.5340 | 3.5846 |
| yjfK | 0.4294 | 3.7685 |
| yjfM | -0.1782 | 3.0266 |
| yjgZ | 2.8882 | 3.3361 |
| yjjB | 0.6138 | 3.3658 |
| yjjP | 0.4738 | 3.9590 |
| ykgB | 0.4550 | 3.4725 |
| ylcH | 0.6785 | 3.9251 |
| ypdF | 1.6239 | 3.3379 |

## Non-CODING regions those affected less by PBS mutants (gene expression in anti-sense direction)

| Co-ordinates | Fold change in gene expression level (Grouped values) | |
|---|---|---|
| | PBS mutants | SBS mutants |
| 357915-358022 | 1.2001 | 3.4466 |
| 499198-499348 | 1.6630 | 4.1688 |
| 527176-527883 | -0.6973 | 3.6324 |
| 527864-528354 | -0.1204 | 4.3537 |
| 528721-528816 | -0.8110 | 3.2165 |
| 2167717-2169751 | 2.2526 | 4.9453 |
| 2663267-2663456 | 2.1295 | 4.1394 |
| 2807516-2807638 | 2.0076 | 4.2569 |
| 2995714-2996850 | 2.9151 | 5.3990 |
| 3621910-3622250 | 0.7082 | 3.1326 |
| 3766662-3766913 | 0.5788 | 3.8835 |
| 3766915-3767279 | 0.0850 | 3.6723 |
| 3767971-3768169 | 0.4993 | 3.5761 |
| 3826689-3826967 | 0.0577 | 3.0610 |
| 3951437-3951500 | 0.4482 | 3.2183 |
| 3955844-3955992 | 1.2378 | 3.6199 |
| 4028995-4029183 | 2.0502 | 3.6462 |
| 4425446-4425716 | 1.5729 | 3.6507 |
| 4425717-4426118 | 0.4648 | 3.2787 |
| 4506699-4506965 | 1.3087 | 3.9089 |
| 4506966-4507826 | 1.5441 | 3.5148 |
| 4571942-4574878 | 1.3672 | 3.0425 |
| 4600882-4601499 | -0.5985 | 3.8964 |
| 4602226-4602332 | 2.0464 | 5.4412 |

Figure S6. List of genes and non-coding regions less affected by PBS mutants compared to that obtained for SBS mutants. Genes belong to the prophages (CP4-6, DLP12, Qin, CP-44) are indicated in colors. Similar to rac prophage, these prophage genes are also not much affected by PBS mutants. Numbers are fold changes in gene expression w.r.t. WT.

A) **Genes affected significantly by PBS mutants**

*Coding genes*

| *Name* | *Fold change in gene expression level ($log_2$)* PBS | SBS |
|---|---|---|
| cysA | 3.402 | 3.89 |
| cysC | 4.516 | 2.126 |
| cysD | 4.457 | 3.162 |
| cysH | 4.624 | 3.837 |
| cysI | 4.878 | 3.961 |
| cysJ | 4.361 | 3.409 |
| cysN | 3.613 | 2.83 |
| cysP | 4.102 | 2.44 |
| cysU | 4.123 | 2.87 |
| cysW | 3.300 | 2.77 |
| fhuF | 3.555 | 4.421 |
| gfcB | 3.333 | 4.536 |
| gfcD | 3.012 | 5.182 |
| pgaB | 3.287 | 3.417 |
| pgaC | 3.203 | 3.893 |
| rho | 3.490 | 2.00 |
| sgcB | 3.809 | 3.40 |
| sgcX | 3.698 | 3.293 |
| yafW | 4.481 | 3.075 |
| yafX | 3.535 | 3.204 |
| yahM | 3.078 | 1.13 |
| yciW | 3.650 | 2.97 |
| ydjN | 3.216 | 2.311 |
| yedK | 3.022 | 2.92 |
| yeeD | 3.171 | 3.737 |
| yeeE | 3.506 | 3.495 |
| yjeF | 3.999 | 5.07 |
| yjhP | 3.244 | 2.84 |
| yjhQ | 3.031 | 2.76 |
| yjjZ | 4.297 | 1.4 |
| ykfG | 4.506 | 2.623 |
| ykfH | 3.980 | 2.501 |
| ykfI | 5.350 | 3.17 |
| ypjJ | 4.238 | 1.506 |
| yqaA | 3.314 | 4.14 |
| yqaB | 4.576 | 3.65 |

*Non-coding regions*

| *Region (nt)* | *Fold change in gene expression level ($log_2$)* |
|---|---|
| 1285750-1285931 | 4.462 |
| 1922994-1923131 | 3.060 |
| 2008514-2008623 | 3.392 |
| 261981-262373 | 3.170 |
| 262374-262436 | 5.773 |
| 262437-262551 | 4.130 |
| 264768-264843 | 3.393 |
| 266000-266191 | 3.405 |
| 2815526-2816982 | 3.366 |
| 4529675-4530072 | 3.305 |
| 4530073-4530333 | 3.355 |
| 4603687-4603826 | 6.537 |
| 497044-497278 | 3.635 |
| 688237-688565 | 3.485 |

B) **Flanking genes of some of the non-coding regions upregulated by PBS mutants**

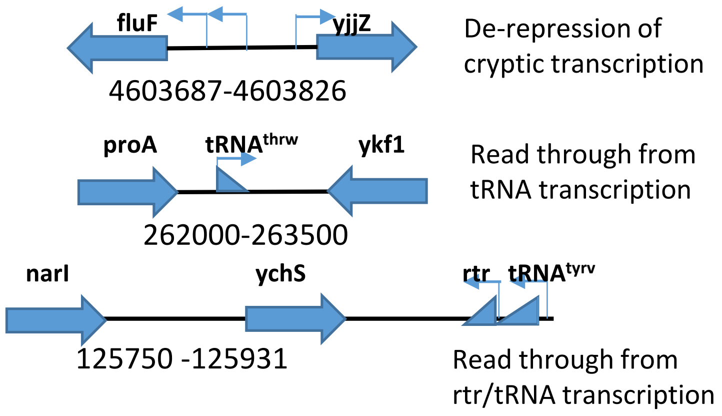


Figure S7. Genes and non-coding regions those are severely affected by PBS mutants.

A)

## *ybcK region*

```
GTTGATTTGT GCCGGTGTGT TGATTATTAA TTTATTGTCA CGAAGCACAC CACATTAAAA TAATTTGTTT CTAAACGACT
AAAATATGGA GGCTCTTATA TTTATATGAG CCTCGTTTTA TGCTTTTTGT TAATGTCTTT ATTTTTTATG TATTCTTTTG
TGCTTTCAAG ATTATGGCGT AAGAAAATTG CAATACGATT ATTGTTGTAT ATTCAAGATA ATGTGACCTT AATTGTCTTT
TTAAATAAAA AATAAACAAA AATTATATCC CACCACTAAG GTTTATAAAA GCATACGTTA GCAGGTGTCA CCATGAAAAA
AGCCATAGCA TATATGCGAT TTTCATCACC AGGTCAGATG TCTGGCGACT CATTAAACCG ACAGAGAAGA CTTATTGCTG
AATGGTTAAA GGTAAATAGT GATTATTATC TTGATACCAT AACATATGAA GATTTAGGAT TAAGTGCATT CAAAGGAAAG
CATGCACAAT CAGGAGCTTT TTCGGAATTT TTAGATGCTA TAGAGCATGG TTATATATTG CCAGGAACTA CATTGTTAGT
TGAAAGTCTG GACAGACTTT CAAGAGAAAA AGTCGGTGAA GCGATTGAAC GTCTGAAATT GATTTTGAAT CACGGTATTG
ATGTTATAAC TCTTTGCGAC AATACAGTCT ATAATATTGA CTCTTTGAAT GAGCCATATT CATTAATAAA AGCCATACTT
ATAGCACAAA GGGCAAATGA AGAAAGCGAG ATAAAGTCAA GTCGGGTTAA ATTATCATGG AAGAAAAAAC GGCAGGATGC
ACTGGAATCA GGTACGATTA TGACGGCGTC TTGTCCGAGA TGGCTCTCCT TAGATGACAA AAGAACGGCT TTTGTTCCAG
ACCCCGACAG GGTGAAAACT ATTGAGCTAA TTTTTAAACT CAGGATGGAA AGGCGCTCAT TGAATGCAAT AGCCAAGTAT
TTAAATGATC ATGCTGTAAA GAATTTCTCA GGAAAAGAAA GTGCATGGGG ACCTTCTGTA ATTGAAAAAT TATTAGCGAA
TAAAGCTCTG ATAGGTATTT GCGTACCTTC ATATCGTGCA AGAGGGAAAG GGATAAGTGA AATCGCTGGC TATTATCCCA
GAGTCATATC AGATGATTTG TTTTACGCTG TACAGGAAAT TCGGTTGGCA CCTTTTGGTA TTAGCAATAG TAGCAAGAAT
CCTATGCTAA TAAATCTACT TCGAACAGTT ATGAAGTGTG AGGCTTGTGG TAATACCATG ATTGTTCATG CGGTATCTGG
AAGTTTGCAT GGCTATTATG TTTGTCCGAT GAGAAGATTA CATCGATGTG ACAGGCCATC AATAAAAAGA GATTTGGTTG
ATTATAATAT CATTAATGAA TTGCTTTTTA ATTGTAGCAA AATTCAACCA GTTGAAAACA AGAAAGATGC TAATGAAACT
TTAGAGTTAA AAATTATTGA GCTTCAGATG AAAATTAATA ATTTAATCGT TGCATTGTCT GTCGCGCCTG AAGTTACCGC
RS848
TATAGCAGAG AAAATAAGAC TATTAGATAA GGAATTACGA AGGGCTTCGG TATCATTGAA AACTTTGAAG AGTAAAGGTG
                                                     RS849
TAAATTCATT CAGTGATTTT TATGCTATTG ACTTAACCAG TAAAAATGGA CGAGAGTTAT GCCGTACACT TGCCTATAAA
ACATTCGAAA AAATCATAAT TAATACGGAT AATAAAACCT GTGATATCTA TTTTATGAAT GGCATTGTTT TTAAACACTA
TCCTTTAATG AAAGTAATAT CCGCCCAGCA GGCGATAAGT GCTCTCAAAT ATATGGTTGA TGGTGAGATT TATTTCTAAA
TAATGATCTC GGATTTTAAG TTATGCTATG GTGATAAAGT GCAAGACAGA ATTAATTATC TTTGACGAAA CTTAATGGGT
```

σ70 promoter

*ybcK*

Figure 8A. Different elements of *ybcK* are indicated. Untranslated region is shown in yellow. Transcription start sites are shown by bent arrows. Primer pair RS848/849 was used for RT-PCR reactions to measure *in vivo* transcriptions described in figure 3E. It is likely that the untranslated region contains the Rho-loading site(s) and the termination occurs inside the gene.

## ***yagN-M region***

```
          RS1083
TTACGAAAAT GGCACGAGAA AATTGAGACA TGGATCTTAA ATGAAGCAGG TATTACCATA AAAAACAACG TTGATATGCG
TTGATTCCAT TAAAAATCAA CATATTACAA AATATCATCA ACTATTGATC AAGATAGATT TTCATGTATC GTAATACACA
GTTTAGTCAA TGATACAGCA ACTACACAGG AGATAAGCCA ATGGCAACCC CAGCAACTGT ATCCATAGAA CCCACTCTGG
CAGCTATCAG AGCTCGCTGG TGTATTAATT CAAGTAAAAC AACTCAATCC TTTAACGATC CTGCGTCCAT GGAAGAGGTT
GTCGAGTATC TCAAAGGAAC ATACTCAGCT CTTCGCAAGT CTGTCGCATG CGCCAAACTG AAAATTTTAC ATCTTAAACA
AAGAATGCAA AATGCTACTA ACTTTCTCGC GCGTCTGATG TCATGTAAAA ATCAGGCATC CAGATCGCAT CACAGTACGG
CTAAATCAGC TAAAAGTGCC TTATCATCAG ATTCAGGTGA TGGTAGTGAC CCCGACCCCG AGCCCGAAAC GTTTCCTTCT
GCCTTCATTA CTACCCCTAC TAATTCAATA ATGCTTAAAG CTTTCTTTGC CAATATCTCA ATCACTGAGG TGGCAAAATG
AGCGCATTCA AACTCCCGGA TACATCTCAA TCACAGCTCA TTTCAACAGC TGAGTTAGCT AAAATCATTA GCTACAAATC
TCAAACCATT CGTAAATGGC TTTGTCAGGA CAAATTGCCT GAGGGGCTAC CTCGCCCAAA ACAAATCAAT GGCCGCCATT
ACTGGTTACG TAAAGATGTC CTCGATTTTA TAGATACATT TTCTGTACGA GAAAGTCTGT AATAAATTAC AGATTTAATT
TTATTGATTT ATAGCGATGT TGCCCCGAGA AAAATGGGGC AACACTGAGA AATTTCAGAT AGTAGTTTTA TATTGAGATA
ACAAAGAGGT TTCCTTAAAA ATGTCTAATA GTGTTACTAA TTTTGAGATG AGCAGCGTTC TACCAGGAAA AAAACCTTGT
               RS1084
CAAGGCAAAA ACAATGAGTC ACAGGTAGTA CAGACTACTC CCATAAAAAA ACACTCAGTC ACGTTCAAAA ATCAATCTTC
ATTAGGAGTA ATTGATCATT ATGCCAGACT AACAAATAAA TCTCACTCTT CCGTAATAGC GGAAGTTGTG GATTTGGCTA
TCCCTATATT AGAAAAATGC AATCGTCATA ACTGGTCAAT AAATGAAATA AAAAATGACC TGTTAAAGTT CTCTATAAAA
GAAAGCATCA ATCGAAGCCG AGGTAAAACA GAAGTAACTC TGGAAGAGTA CTGTTCGTTA ATCTGGAAAA CGAACATCAT
GAGTCCATTA AAAATCCCCA TTGCAGATTA CTTTCAACTG AACGCTAATG ATGAATTCAT GGGGAAAGAT GAAAAAACAG
TTATACGTGA AAGGCTATCC TCGCTAAGGG AAAATTACGA TATGGAAAAA GCCATTTACA TTTACAATCA AAGACATTTT
GATGTAAAGC ATCAAAGTGT CTCAGGATAT TCAAACATTA TTCTTATTCA TAGAACAACC TTTGAGGGTT ATTACTTTGA
TGCCGGGCAG GCTCTACTCT TGTCAACATC CCAATTGATT ATATTCGGGA TAAATGAAGT TCTTAGAAGA AAGGGGATTG
TTATGCCTTA TCCGGTTGTT TGTTGGATTG ATATTTACCA TGTCAATGAA ATGGTGGTTA TGCTGCCAGT GCTCCGCAAA
               RS846
ACAGATGTTT CCAACCGTGT TAATGTACCG GATGACATCA TTATAAACCC ATACTCACAA GAGAGCAGAA CCTAA RS847
```

σ70 promoter

*yagN*

*Intergenic region*

*yagM*

Figure 8B. Different elements of yagN-M operons are indicated. Untranslated region is shown in yellow. transcription start sites are shown by bent arrows. Primer pairs RS1083/1084 and RS846/847 were used to make DNA templates for *in vitro* transcription by PCR and for qPCR reactions, respectively.

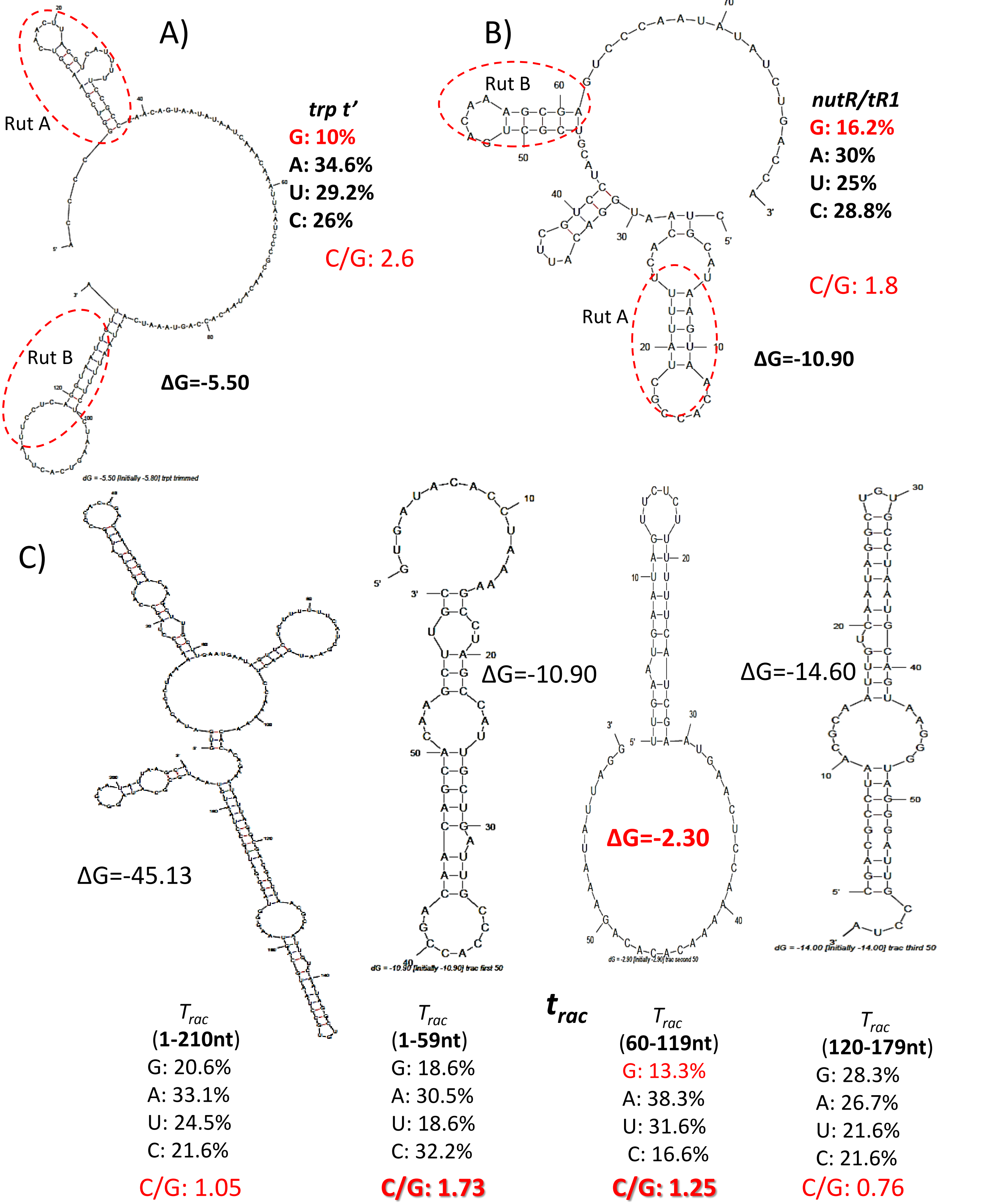


Figure S9: The predicted secondary RNA structures of rut site regions of different terminators determined by the M-fold program. The terminators *trpt'* and *tR1* are shown in A) and B). Rut sites are indicated by dotted ovals. Compositions of the bases in each of these regions are indicated together with the free energy (ΔG)of the secondary structure formation. C) The 210 nt untranslated region preceding the *racR* region is folded as a whole and part wise. The part, 60-119nt has the lowest ΔG and also is G-poor. The region 1-59 nt has also G-poor sequence. And hence, these regions of the untranslated part of $t_{rac}$ would have the Rho-loading sites.

## M-Fold plots of 151nt ybcK untranslated region

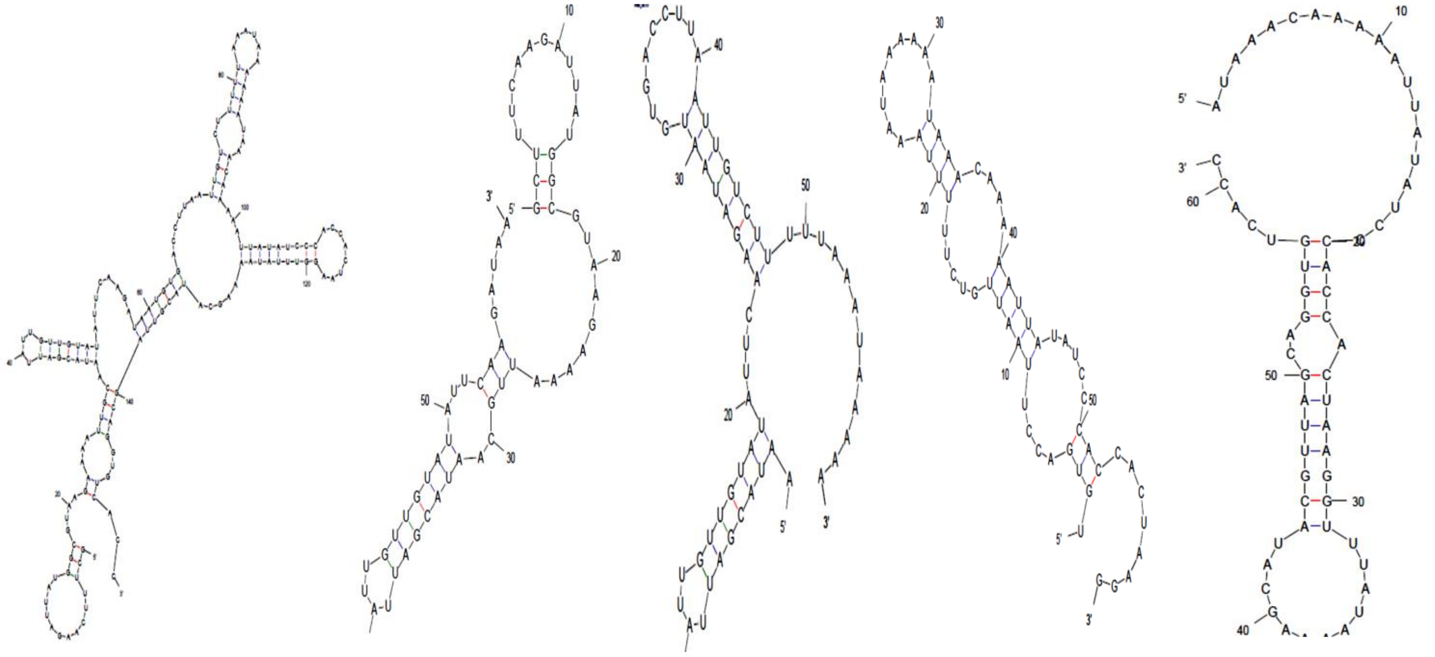

| (1-151nt) | (1-60nt) | (31-90nt) | **(61-120nt)** | (91-151nt) |
|---|---|---|---|---|
| ΔG= -13.94 | ΔG= -5.90 | ΔG= -9.10 | **ΔG= -0.20** | ΔG= -8.00 |
| C/G= 1.00 | C/G= 0.54 | C/G= 0.71 | **C/G= 2.00** | C/G= 1.62 |

Figure 10A. The region (in red), 61-120nt, has the lowest free energy of secondary structure formation and high C/G ratio, and hence is likely to have Rho-loading site(s).

## M-Fold plots of 87nt untranslated region of yagN-M operon

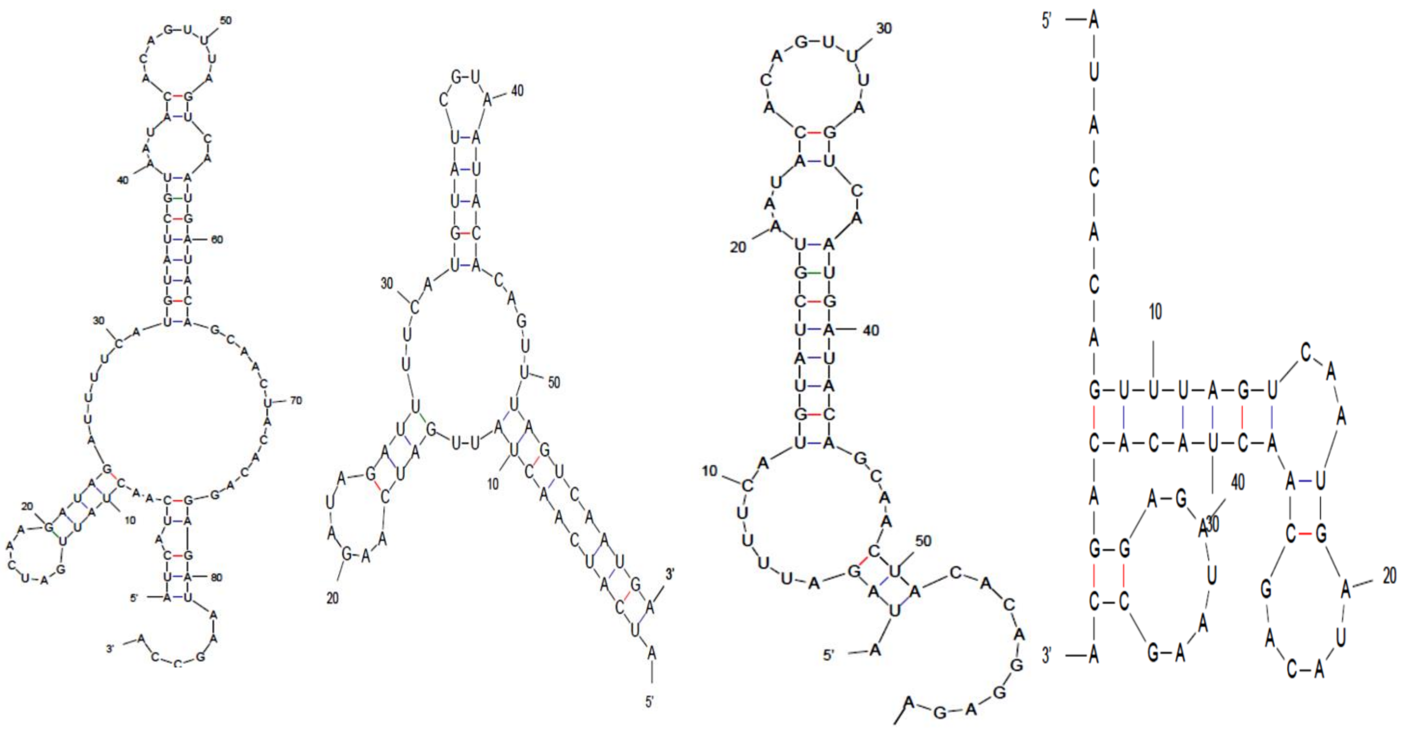

| (1-87nt) | (1-60nt) | (21-80nt) | **(41-87nt)** |
|---|---|---|---|
| ΔG= -6.22 | ΔG= -4.13 | ΔG= -6.30 | **ΔG= -2.40** |
| C/G= 1.23 | C/G= 1.12 | C/G= 1.00 | **C/G= 1.25** |

Figure 10B. The region (in red), 41-87nt, has the lowest free energy of secondary structure formation and high C/G ratio, and hence is likely to have Rho-loading site(s).

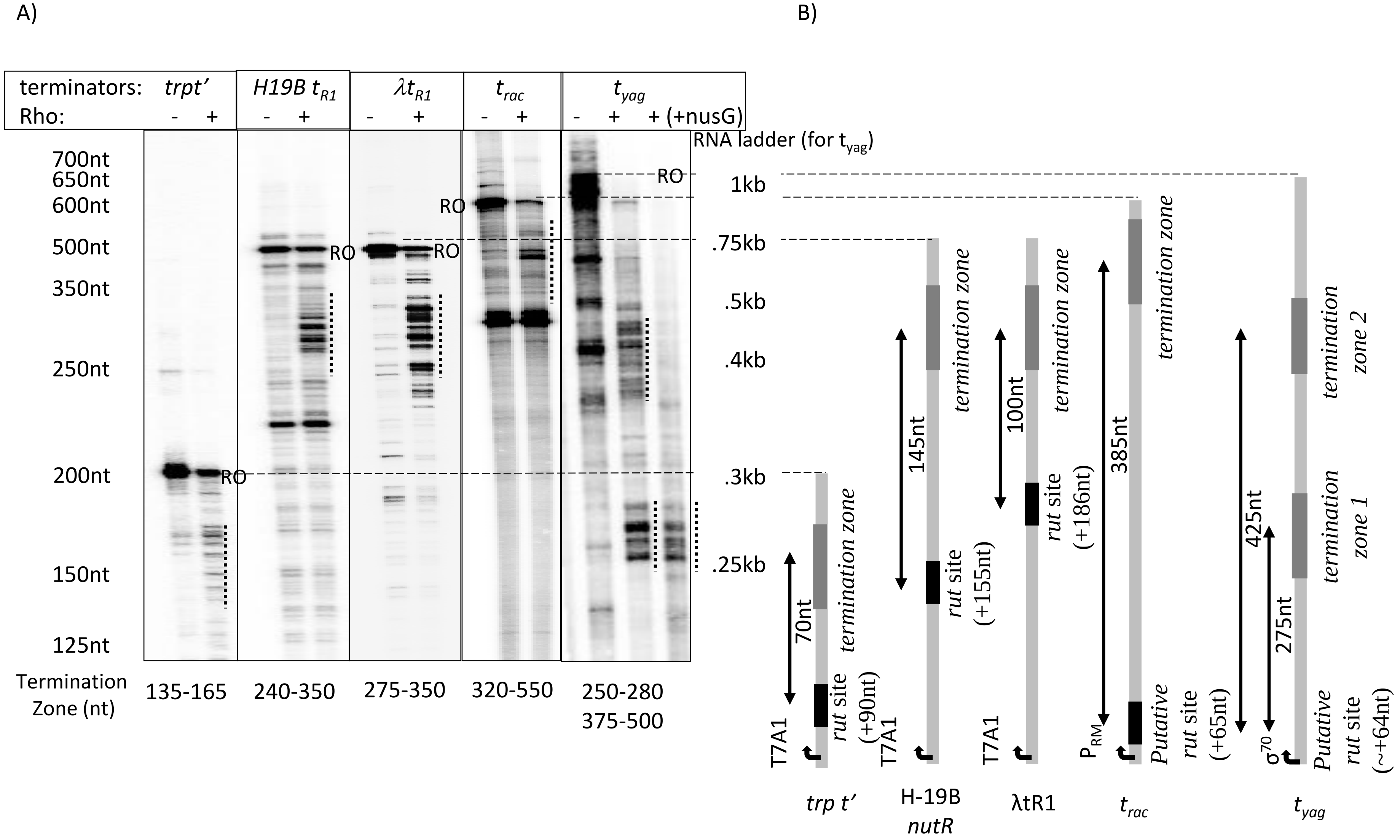


**Figure S11**: **A)** *In vitro* termination assays on different terminators. Termination zones for each are indicated by dashed vertical lines, and their mean distances from the start site are indicated below the autoradiograms. $t_{yag}$ has two termination zones. Last lane of this panel is in the presence of NusG. Size of the transcripts of the $t_{yag}$ panel aligns to the RNA ladder. It should be noted that like $t_{rac}$, $t_{yag}$ is also highly NusG-dependent, as indicated from the early termination bands. **B)** Cartoons showing the mean distances between the *rut* site and the termination zones for different terminators. The positions of *rut* sites and the termination zones are w.r.t. the transcription start site. DNA template ends are roughly aligned with the position of the run-off (RO).

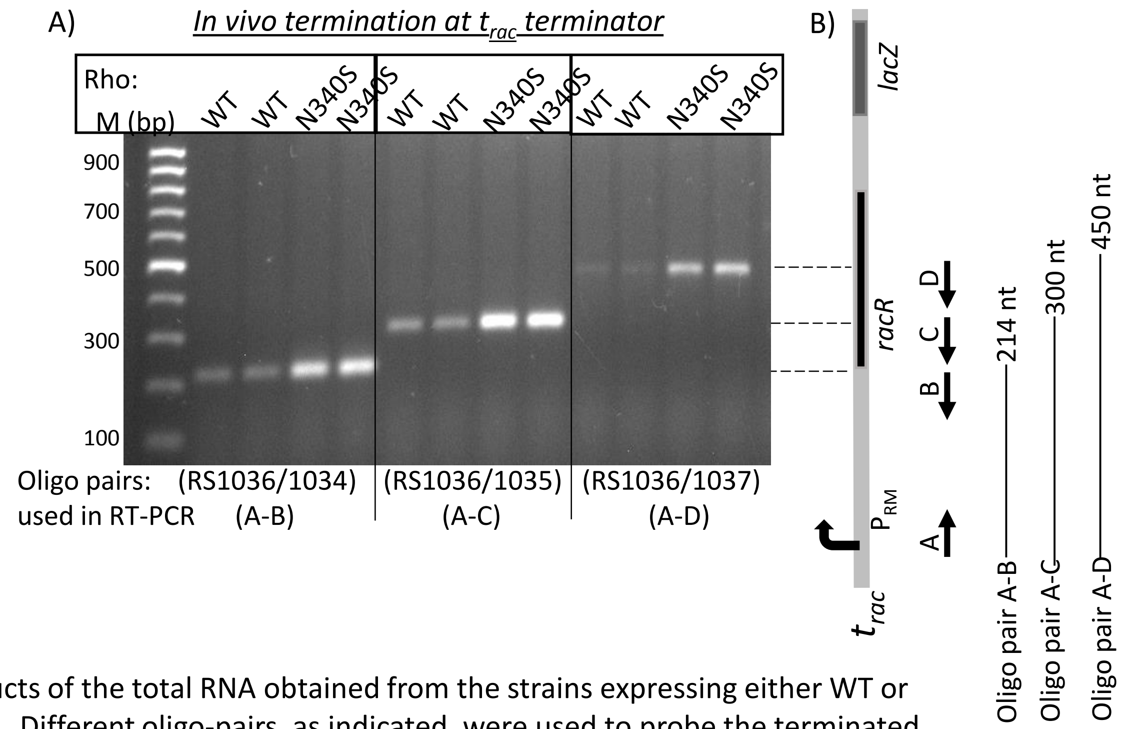


**Figure S12**. **A)** RT-PCR products of the total RNA obtained from the strains expressing either WT or N340S Rho mutant (RS1428). Different oligo-pairs, as indicated, were used to probe the terminated RNA products initiated from $P_{RM}$ promoter. Two reactions were loaded for each case. These cDNAs were from terminated RNA because the amount of the product is much higher in the presence of N340S mutant. **B)** Cartoon showing the positions of various oligos used and the lengths of cDNA products aligned to different regions of *racR* region. This indicates that the major *in vivo* termination events occurred inside the *racR* region.

Table S1: List of oligos used.

| Oligos | Description |
|---|---|
| RS83 | ATAAACTGCCAGGAATTGGGGATC; upstream oligo of pTL61T vector sequence, 5' biotinylated |
| RS139 | TTAATACGACTCACTATAGGGAGATCGAGAGGGACACGGGCG; T7 promoter fused to the start site of T7A1 promoter |
| RS147 | GCGCGCGGATCCCCCATTCAAGAACAGCAAGCAGC; lambda *TR1* reverse primer with BamHI site. |
| RS401 | GCGCGCGTGGTGCAACGGGCGCTGGG; *lacZ* specific FP for qPCR |
| Rs402 | GCGCGCCAACTCGCCGCACATCTG; *lacZ* specific RP for qPCR |
| RS404 | *GAATTGTGAGCGCTCACAATTC*GGATGCCAGACCGCGCTGGGTAAGCG; RP with 5'-lacO fusion, used to generate roadblock downstream of rut sites of for *T7A1-H19B TR1* template |
| RS555 | CTG CTT CAG GAT GGC AAA AAT AAT GTC C ; internal RP (123-96bp) of *rho* gene used for checking rho deletion by PCR |
| RS845 | *GAATTGTGAGCGCTCACAATTC*TTAGCGCCTCGGGCTGGCTA; RP with 5'-lacO fusion used to generate roadblock on $P_{RM}$-*racR*/$t_{rac}$template |
| RS846 | CCTTATCCGGTTGTTTGTTG; *yagM* specific FP for qPCR |
| RS847 | TTAGGTTCTGCTCTCTTGTG; *yagM* specific RP for qPCR |
| RS848 | CCTGAAGTTACCGCTATAGC; *ybcK* specific FP for qPCR |
| RS849 | CTCTCGTCCATTTTTACTGG; *ybcK* specific RP for qPCR |
| RS852 | AATCGCAAAGCAATCTTGGT; *gshA* specific FP for qPCR |
| RS853 | GCAACACGTTGCTGTTGATT; *gshA* specific RP for qPCR |
| RS854 | CTGGTAACAAACCAGAGTGG; *rpoC* specific FP for qPCR |
| RS855 | TCAGACGGTTGTTACGGTTA; *rpoC* specific RP for qPCR |
| RS955 | *GAATTGTGAGCGCTCACAATTC*TTAGGTATTCGTTAAGCCCC; RP with 5'-lacO fusion, used to generate roadblock on $P_{RM}$-*racR*/$t_{rac}$template |
| RS956 | *GAATTGTGAGCGCTCACAATTC*TTAGGGCGCGAACATCGTGG; RP with 5'-lacO fusion, used to generate roadblock on $P_{RM}$-*racR*/$t_{rac}$template. |
| RS963 | AACAATGTCGACTTAACCTGAACGACGACGATTAC; *rhlB* specific primer for checking *rho* deletion by PCR |
| RS995 | *TTAATACGACTCACTATAGGGAGA*GTGATACACCTAAAAGCCTAGCC; T7 promoter fused to the start site of $P_{RM}$ promoter. |
| RS1034 | CATTGCTTAATATTCTCCTATGCGC; RP for RNA preparation on RS1352 |
| RS1035 | TGCGACCTCCGCCTTTGATTTGACGG; RP for RNA preparation on RS1352 |
| RS1036 | GAGGTGATACACCTAAAAGCCTAGCC; FP at transcription start site of T7A1 of pRS1352 |
| RS1037 | TTGGTATGGGGTATTCGTTAAGCCCC; RP at *racR* gene of pRS1352. |
| RSRK-1 | GTTTTCCCAGTCACGAC; reverse primer in *lacZ* gene. |
| RK23B | TGGAGTTCCAGACGATACG; reverse oligo to generate *T7A1-H19B/TR1* terminator template |
| RS1083 | GGCACGAGAAAATTGAGACATGG; FP in *yagN* region, to be used to prepare $t_{yag}$ terminator template |
| RS1084 | CTACCTGTGACTCATTGTTTTTGCC; RP in *yagM* region, to be used to prepare $t_{yag}$ terminator template |

Table S2: β–galactosidase activities of different lacZ fusions under different conditions.

| *NusG alleles* | $T_{R1}$*-LacZ β–galactosidase activity* | $T_{R1}$ *–trpt'-LacZ β–galactosidase activity* | $T_{rac}$*-LacZ β–galactosidase activity* |
|---|---|---|---|
| WT | 259.4 ± 5 | 20.2 ± 0.08 | 61 ± 0.5 |
| V160N | 634.3 ± 60 | 34 ± 0.8 | 2421 ± 46 |
| G146D | 461.3 ± 9 | 49 ± 0.9 | 1865 ± 64 |
| L158Q | 460 ± 10 | 48± 2 | 2766 ± 75 |
